# A Critical Evaluation of the Hybrid KS DFT Functionals Based on the KS Exchange-Correlation Potentials

**DOI:** 10.1021/acs.jpclett.4c01979

**Published:** 2024-10-02

**Authors:** Vignesh Balaji Kumar, Szymon Śmiga, Ireneusz Grabowski

**Affiliations:** Institute of Physics, Faculty of Physics, Astronomy, and Informatics, Nicolaus Copernicus University in Toruń, ul. Grudzia̧dzka 5, 87-100 Toruń, Poland

## Abstract

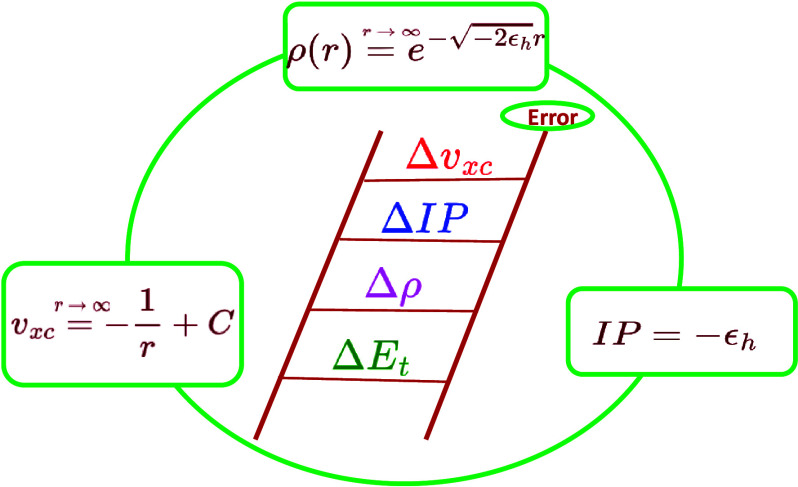

We have developed a critical methodology for the evaluation of the quality of hybrid exchange-correlation (XC) density functional approximations (DFAs) based on very fundamental quantities, i.e., Kohn–Sham (KS) XC potentials, self-consistent electron densities, first ionization potentials (IPs), and total energies. Since the XC potentials, the primary objects in the current study, are not directly accessible for the hybrids, we calculate them by inverting the KS electron densities. Utilizing this methodology, we tested 155 hybrid DFAs available in the LIBXC library using FCI and CCSD(T) methods as a reference. We have found that a group of functionals produces very decent XC potentials, mainly those with a large mixture of Hartree–Fock exchange. Moreover, the value of IP strongly depends on the XC potential quality. On the other hand, we show that the XC energy is dominated by functional-driven error, which in some cases leads to substantial errors in electronic densities. The study shows new directions for constructing more accurate XC functionals within the KS-DFT framework.

Density functional theory (DFT)^1^ is one of the most popular computational methods extensively applied in chemistry, physics, and materials science calculations. In principle, DFT is an exact theory; however, in practice, for the implementation of Kohn–Sham’s (KS) approach,^2^ it requires an approximation for the exchange-correlation (XC) energy functional. Hundreds of nonempirical and semiempirical density functional approximations (DFAs) have been developed in the past six decades, presenting different levels of complexity and quality.^3^ Among them, so-called hybrid functionals are common in standard DFT applications and have proven practical in KS-DFT calculations,^4^ being the best compromise between accuracy and efficiency. In general, hybrid XC functionals (*E*_*xc*_^HYB^) are a mixture of a fraction of the Hartree–Fock (HF) exact exchange energy (*E*_*x*_^EXX^) with some parts of semilocal exchange and correlation functionals, usually at LDA, GGA, and meta-GGA levels.^5^ DFT hybrids introduced by Axel Becke in 1993^6^ can be theoretically justified with the adiabatic connection formula^7−10^ and generalized KS (GKS) framework.^11−13^ The popularity of hybrid functionals reflects their essential features, i.e., systematically higher predictive accuracy for several properties,^3,14^ reduction of the self-interaction errors (SIE), and approaches to address the KS derivative discontinuity^15^ allowing to overcome the bandgap partially or the charge transfer excitation problem.^16−18^ In addition, to ensure the proper behavior of the exchange potential at the long-distance limit, the range-separated (RS) class of hybrids^19^ was successfully developed.

By definition, the KS XC potential (*v*_*xc*_) is obtained as the functional derivative of the XC energy functional (*E*_*xc*_) with respect to the density (ρ)
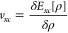
1

We note that the hybrid class of KS functionals depends directly on KS orbitals via the nonlocal HF (exact) exchange energy term and kinetic energy density^20^ utilized by meta-GGA hybrids to define these functionals. For this reason, to remain entirely in the KS framework, one needs to employ the optimized effective potential (OEP) method^21−23^ to compute the XC potential in eq 1. In practice, however, to avoid difficulties related to the OEP method,^24−26^ the hybrid functionals are realized within the GKS framework^11−13^ with nonlocal XC potential. Nonetheless, as pointed out in many studies,^27−30^ the GKS and full KS realizations of hybrids give practically the same accuracy for ground-state properties, differing only for quantities involving excited states.^31^

For this reason, the KS XC and correlation potentials are not usually the objects of study in the process of developing new XC functional approximations. We remark, however, that these potentials are well-defined quantities that directly enter the KS equations. As was pointed out in a recent study,^30,32^ the hybrid functionals can provide reasonably accurate local XC^32^ potentials and densities^30^ obtained within the KS scheme realized via the OEP method^30^ and the inverse procedures.^32^ Moreover, it was shown in ref (30) that the correlation functionals (*E*_*c*_^HYB^), which can be defined for any hybrid XC approximation as *E*_*c*_^HYB^ = *E*_*xc*_^HYB^ – *E*_*x*_^EXX^, give very physical correlation potentials, capable of describing the quantum shell oscillations of the high-level reference data for atoms and molecules correctly.

The present study is devoted to a generalized assessment of the most popular hybrid XC functionals available in the LIBXC library.^33^ To this end, we have tested 155 hybrid functionals, mainly focusing on their XC and correlation potentials, which are usually underestimated and even ignored in DFT functional development. Certainly, there are some exceptions, e.g., *ab initio* DFT,^26,34−36^ correlated orbital theory (COT),^37^ and Handy and co-workers’ work.^38−40^ Here, we show how significant and influential the KS XC potential is for investigating the properties and quality of existing XC functionals and developing new DFT functionals. In addition, our evaluation also focuses on the fundamental KS-DFT features: total energies, electron densities, and ionization potentials, which are the standard quantities used in the KS-DFT assessment process. This allows us to reveal and confirm some essential relations, e.g., the correlation between the quality of the XC potential and the accuracy of ionization potentials for a given hybrid XC DFT approximation (DFA). We also analyze density- and functional-driven errors for all hybrid functionals. This work paves the way for further functional development of these types of DFAs by significantly increasing their predictive power.

In our assessment, we have considered 155 hybrid functionals currently available in the LIBXC library^33^ (version 6.1.0.), representing the most important and most frequently used hybrid approximations in practical DFT calculations. All functionals are listed and numbered in Table ST1 in the Supporting Information (SI) file, with the original names kept as indicated in the LIBXC library. To make our overview of hybrids more systematic, within Table ST1, the functionals are organized using three main factors: (i) they are divided into the global and RS classes of hybrids; (ii) within each class, they are grouped according to standard DFT approximations classification, i.e., LDA, GGA, and meta-GGA (MGGA); (iii) and finally, within these groups, the functionals are arranged in ascending order according to a fraction of the nonlocal HF exchange energy contribution in the XC approximation. In the case of global hybrid DFAs, the HF fraction is described by the value of the HF coefficient itself, whereas, in the RS hybrids, it is described by the long-range HF coefficient. Both coefficients are taken directly from the LIBXC library. The same ordering of DFAs is utilized in Figure 2 and Figure 3.

Our primary object in the current study is XC potential, which is computed from a given self-consistently obtained hybrid GKS density matrix, employing the Wu–Yang^41^ (WY) inversion procedure (for more details, see Section 2 in the SI file).

We also calculate the correlation potential as *v*_*c*_ = *v*_*xc*_ – *v*_*HF*_, where *v*_*HF*_ is the local, exchange-only potential obtained from the self-consistently obtained HF density. Additionally, in our assessment, we have also considered a few quantities that are directly accessible from standard self-consistent GKS realization of hybrids, i.e., total energies, electron densities, and ionization potentials computed from the highest occupied molecular orbital (HOMO) energy (*IP* = −ε_*HOMO*_). The comparison of densities, XC potentials, and IPs is critical from the conceptual point of view of KS-DFT theory and the development of new XC approximations. We recall that the asymptotic behavior of the XC potential governs the quality of the first IP, which also impacts the behavior of electron density in the asymptotic tail. The density, in turn, is connected by one-to-one mapping of the Hohenberg–Kohn theorems with XC potential.^1,2^ Thus, all of these quantities are related, as depicted in Figure 1.

**Figure 1:**
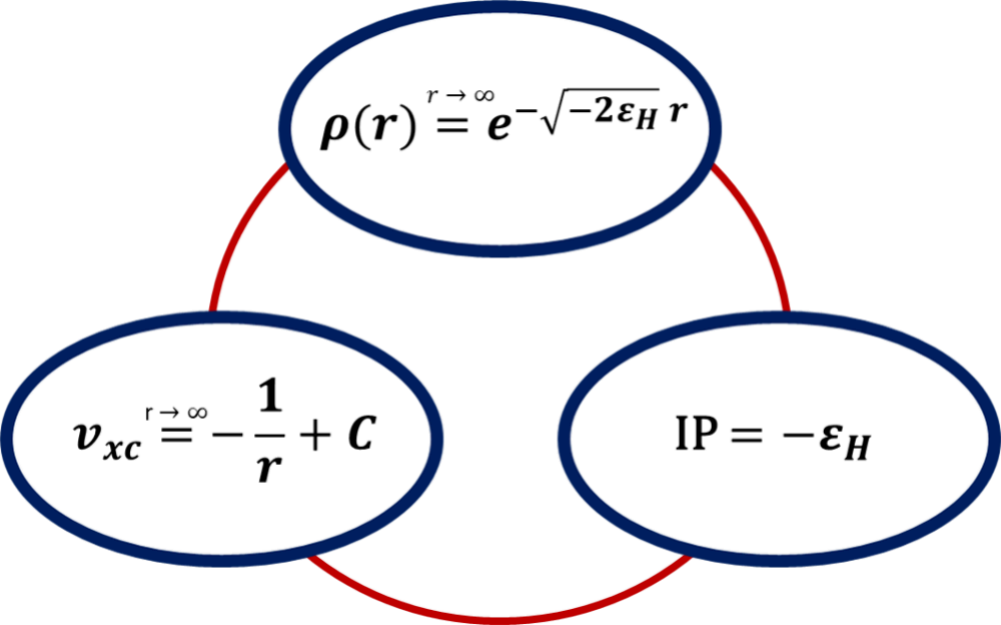
Graphical representation of relations between ρ, *v*_*xc*_ and IP in KS-DFT (ε_*H*_ = ε_*HOMO*_).

To quantify the hybrid DFA comparison, we use a few error measurements,^32^ namely,
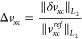
2
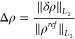
3where *Δv*_*xc*_ and *Δρ* are the relative errors of the hybrid XC potential (*v*_*xc*_) and electron density ρ, respectively, defined using the standard *L*_2_ norm, so, e.g., ∥*δA*∥_*L*_2__ = (∫d**r**(*A*^*ref*^(**r**) – *A*(**r**))^2^)^1/2^, where for a given quantity *δv*_*xc*_ = *v*_*xc*_^*ref*^ – *v*_*xc*_ and *δρ* = ρ^*ref*^ – ρ are computed at every grid point. Because of that, the *Δv*_*xc*_ can give rise to significant errors for hybrids that exhibit differently than −1/*r* asymptotic behavior in the tail of the XC potential.^30^ Section 3 in the SI discusses this issue in more detail. Hence, we consider the *Δv*_*xc*_ error as a good indicator of the quality of XC potentials, mainly in the asymptotic limit. We recall that asymptotic behavior of the XC potential has a significant impact on the quality of the first ionization potential, as well as excitation energies.^19,42,43^ We also calculate the error of the correlation potential *Δv*_*c*_ in analogy to *Δv*_*xc*_ (see eq 2). In addition, to get some insight into how XC potentials behave in the core and valence regions, we define another useful error indicator:
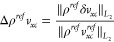
4

Because ρ^*ref*^ decays much faster than *v*_*xc*_ as we move away from the center of the nuclei, we eliminate from *Δρ*^*ref*^*v*_*xc*_ error a large contribution coming from the asymptotic tail and focus on the behavior of *v*_*xc*_ in more energetically important (the core and valence) regions.^32^

Because the total energy and IPs are the single values corresponding to the system, we use the absolute relative error (ARE) in their error evaluation *ΔE*_*t*_ and *ΔIP*
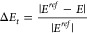
5
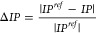
6where *E*^*ref*^ and *IP*^*ref*^ denote the reference total energy and IP, respectively, whereas *E* and *IP* correspond to their hybrid counterparts. The errors given by eqs 2–6 are computed separately for each system and hybrid functional.

The reference energies *E*^*ref*^ and densities ρ^*ref*^ have been calculated at the FCI and CCSD(T) level of theory depending on the considered benchmark set (see below), whereas the very accurate IP-EOM-CCSD^44,45^ method was used as a reference for a first vertical IP. In turn, in the case of reference XC potentials *v*_*xc*_^*ref*^, the WY inverse procedure is employed to obtain the potentials from the FCI or CCSD(T) relaxed density matrixes, depending on the benchmark set.

In our consideration, we use two benchmark sets: (i) FCI benchmark set consisting of systems up to four electrons, i.e., He, H_2_, He_2_, Be, and LiH for which reference data have been obtained at the FCI level of theory; (ii) CCSD(T) benchmark set consisting 16 atomic and molecular systems listed in Table 1 in ref (46) for which reference data have been obtained at the CCSD(T) level of theory. The geometries for molecular systems are taken from ref (46).

As in similar studies^32,47^ utilizing the WY method, the triple ζ quality basis set was used (uncontracted aug-cc-pVTZ^48^) in all calculations, in order to make a comparison on the same footing and to reduce basis set related errors. The same basis set is used for potential expansion to ensure a smooth, balanced solution with the WY inverse method. We underline that the same qualitative behavior is obtained by using the larger basis set, namely, uncontracted aug-cc-pV5Z^48−50^ (see Figure S2 in the SI file). Thus, at this point we conclude that a triple ζ quality basis set is sufficient for the present investigation.

The FCI and CCSD(T) reference electron densities and total energies are computed using the Psi4^51^ program. The reference XC potentials are calculated via the WY scheme using the *n2v* package of the same software.^51,52^ The hybrid XC potentials, in turn, are computed using the same WY inversion procedure with the *n2v* package and PySCF^53,54^ program. We refer the reader to Section 2.1 in the SI file for more information regarding the WY method. The reference IPs are obtained with the IP-EOM module from the PySCF package.

In all GKS DFT calculations, we have employed a Lebedev and Laiko quadrature formula for DFT grid construction^55,56^ that corresponds to level 5 as implemented in the PySCF package. We underline that errors defined by eqs 2–4 have been evaluated on the same DFT grid as used for KS-DFT calculations. We also note that the convergence of these errors has been tested concerning grid size, and the level 5 constructed in PySCF package is sufficiently large for this assessment (see Section 2.2 and Figure S1 in the SI file).

We start our analysis by directly comparing errors defined by eqs 2–6 computed separately for the Be atom and LiH molecule, w.r.t. FCI references for all 155 hybrid DFAs. We report these in Figure 2. The *X*-axis corresponds to various hybrid functionals numbered and grouped as Table ST1 indicates. To make the analysis easier, we highlight the main groups of DFAs by using color coding.

**Figure 2:**
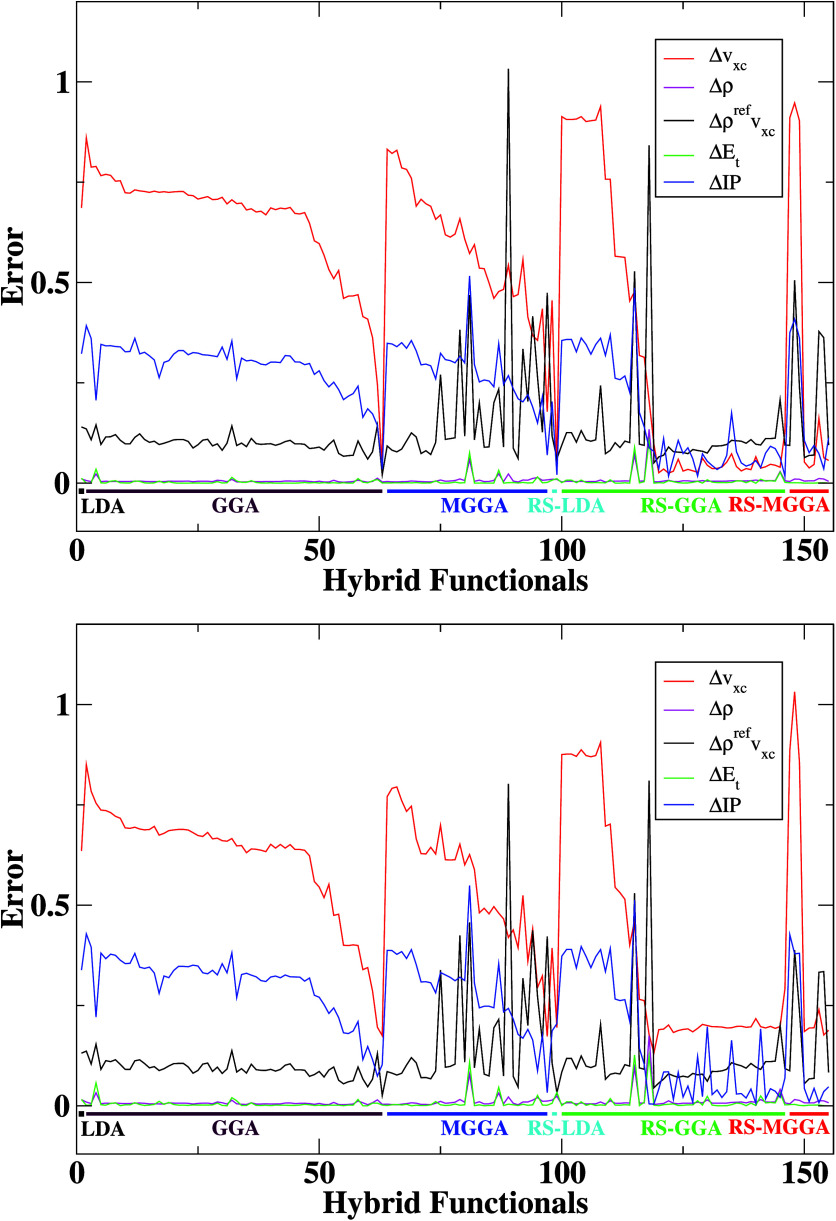
Errors defined by eqs 2–6 for the Be (top) atom and LiH (bottom) molecule with the FCI reference. The IP EOM-CCSD method is used as a reference to calculate IP error. The *X*-axis corresponds to various hybrid functionals numbered and grouped according to Table ST1.

One can note that the error behaviors are almost identical for both systems, qualitatively and quantitatively, and among all of the functional groups. This is also the case for other systems analyzed in our research. For credibility purposes, we also report, in Figure S3 in the SI, the error plots for the Ar atom and HCl molecule calculated with respect to the CCSD(T) reference. This is the first important finding of the present study.

Similar behavior can also be noted for averaged errors computed separately for the whole FCI and CCSD(T) benchmark sets, as presented in Figure 3. Here, we see that the qualitative behavior of average errors is very similar, despite the size of the benchmark set and reference data. Therefore, in further analysis, we will focus only on average errors calculated concerning the CCSD(T) reference data (CCSD(T) benchmark set) to describe the main trends in the hybrid DFAs performance. These data are collected for all hybrid functionals in the external repository (10.18150/V4XZSF),^57^ together with the HF fraction coefficients. This can be useful to the reader for checking, e.g., different types of errors for individual functionals and for making their additional analyses.

**Figure 3:**
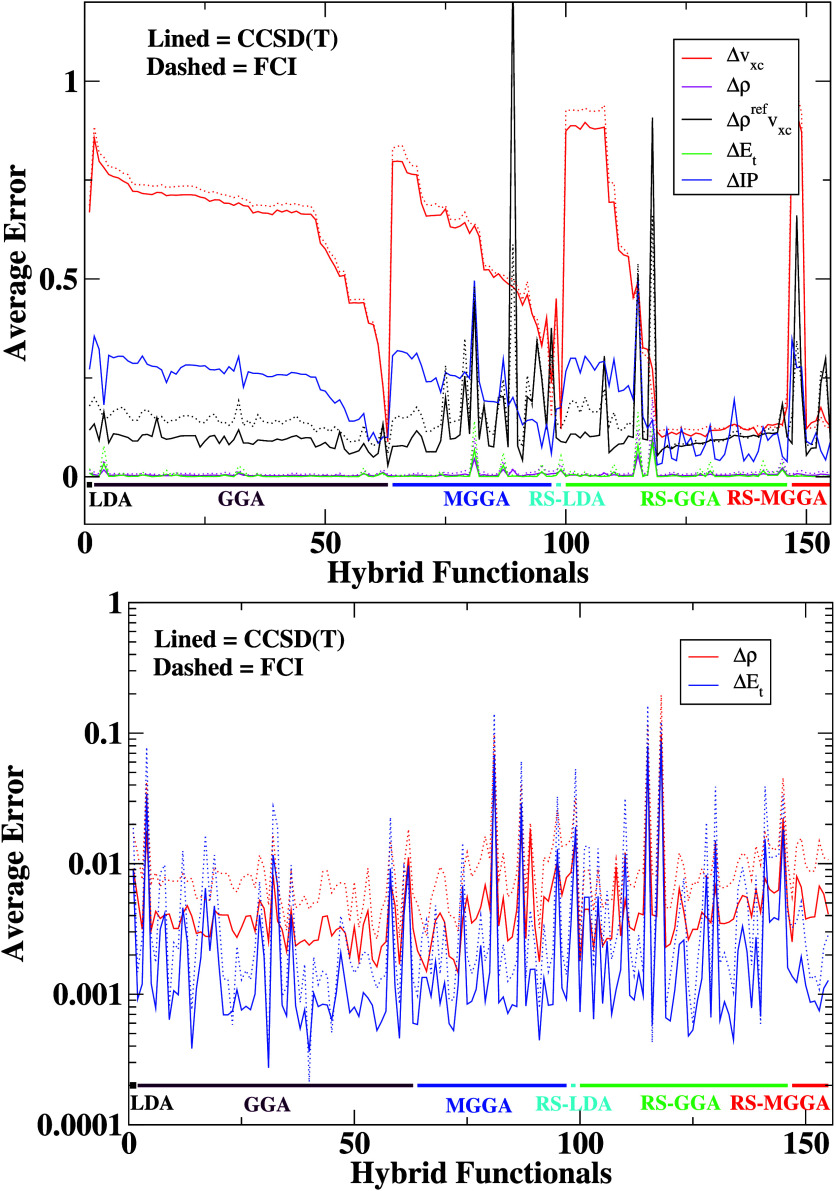
Top panel: Average errors (see the text) were calculated for the FCI and CCSD(T) benchmark sets. The IP EOM-CCSD method is used as a reference to calculate IP error. Bottom panel: As in the top panel but scaled up for total energy and density errors. Please note the logarithmic scale on the *Y*-axis at bottom panel. The *X*-axis corresponds to various hybrid functionals numbered and grouped according to Table ST1.

By analyzing the error plots in Figure 2 and Figure 3, one can note a considerable deviation between the sizes of the different types of errors (*Δv*_*xc*_, *Δρ*, *Δρ*^*ref*^*v*_*xc*_, *ΔE*_*t*_, *ΔIP*). In most cases, the total energy error (*ΔE*_*t*_) is the most minor, usually three or four orders smaller than the XC potential error. This is not surprising, because good performance for energetics is usually the primary concern of XC functional developers. We note that most functionals have been parametrized regarding several types of energy qualities or physical constraints related to energetics (e.g., second-order gradient expansion,^58^ Lieb–Oxford bound,^59,60^ etc.), and only very few of them have been developed to have correct XC potentials^61^ or IPs.^62,63^

We also observe that the density error (*Δρ*) is very similar in magnitude to the total energy error (*ΔE*_*t*_) for all hybrids. Therefore, we present total energy and density errors on an additional separate plot (bottom panel of Figure 3). This can also be observed in Figure 4, where we analyze *ΔE*_*t*_ and *Δρ* errors together. One can note that for both errors many hybrid DFAs yield accuracy between HF and MP2 methods. It confirms the well-known behavior of the KS DFT hybrids versus the *ab initio* methods. In particular, none of the hybrid DFAs exceeds the MP2 method for the averaged *Δρ* error, which agrees with the findings from ref (64) (see Table 1).

**Figure 4:**
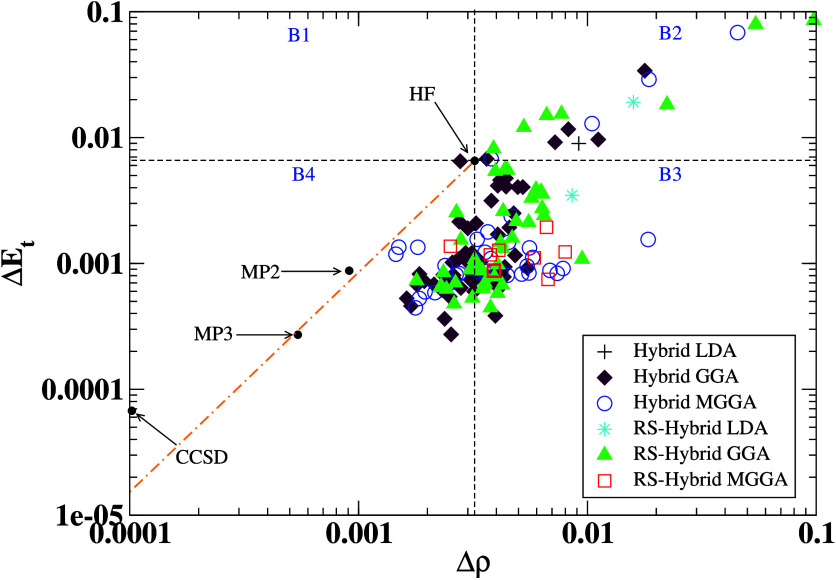
Average *Δρ* error vs average *ΔE*_*t*_ error for the CCSD(T) benchmark set. Note the logarithmic scale used for both axes. The orange (dash-dotted) line in block B4 indicates the ideal error decrease trend.

**Table 1:** Three Hybrid XC DFAs with the Largest Average Errors Calculated for the CCSD(T) Benchmark Set

Error Type	XC Functional	Type	Error
Δ*E*_*t*_	CAM-O3LYP	RS-GGA	0.0854
	WHPBE0	RS-GGA	0.0795
	X1B95	MGGA	0.0683
			
Δρ	CAM-O3LYP	RS-GGA	0.0974
	WHPBE0	RS-GGA	0.0543
	X1B95	MGGA	0.0453
			
Δ*v*_*xc*_	MN12-SX	RS-MGGA	0.9206
	HJS-B97X	RS-GGA	0.8959
	HJS-PBE-SOL	RS-GGA	0.8875
			
Δρ^*ref*^*v*_*xc*_	MN15	MGGA	1.2917
	CAM-O3LYP	RS-GGA	0.9080
	MN12_SX	RS-MGGA	0.6605
			
Δ*v*_*c*_	MN12-SX	RS-MGGA	13.4627
	HJS-B97X	RS-GGA	13.2353
	HJS-PBE-SOL	RS-GGA	13.1643
			
Δ*IP*	WHPBE0	RS-GGA	0.4959
	X1B95	MGGA	0.4957
	MPWLYP1M	GGA	0.3552

On the other hand, a large group of hybrids yield much worse densities than those given by the HF method. In particular, one can divide the Figure 4 area into four interesting regions by taking the HF results as a reference point (*Δρ*^HF^ = 0.0032, *ΔE*_*t*_^HF^ = 0.0065). In the B1 part for which *Δρ* < *Δρ*^HF^ and *ΔE*_*t*_ > *ΔE*_*t*_^HF^ we do not register any functionals, which indicates that none of the existing hybrid XC DFAs can generate a simultaneously good quality of densities (better than the HF one) with poorer performance for total energies (worse than the HF method). In the B2 region (*Δρ* > *Δρ*^HF^ and *ΔE*_*t*_ > *ΔE*_*t*_^HF^), in turn, one can note quite a few DFAs (18 cases). In particular, in this block, we can find the group of energetically (*ΔE*_*t*_) worse-performing hybrid functionals, namely, the CAM-O3LYP, WHPBE0, and X1B95 (see also Table 1), which are placed in the right top corner of Figure 4. Interestingly, these three functionals also yield the largest density error values *Δρ* (see also Table 1), indicating the possible correlation between *ΔE*_*t*_ and *Δρ* errors for worse-performing functionals. This can be related to a large self-interaction error build-in in these DFAs, which is then transferred to the electron density via a self-consistent DFT procedure. In other words, this shows that, if the functional is very bad for energetics, it will also most probably lead to large density errors. The third block (B3) contains a large number of functionals (82 cases) for which *Δρ* > *Δρ*^HF^ and *ΔE*_*t*_ < *ΔE*_*t*_^HF^. Here we can find, e.g., popular B3LYP,^65^ HSEsol,^66^ or QTP17^63^ DFAs. Energetically, these hybrid DFAs, by definition, should include correlation effects that are beyond the HF picture. On the other hand, the same functionals produce electron densities that perform worse than the HF method. To analyze that in more detail, we have decomposed the total energy error into the density-driven (DD) and functional-driven (FD) parts^47^ computing the absolute relative DD and FD errors separately as
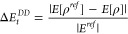
7and
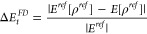
8respectively, for the CCSD(T) set. Figure S4 and S5 in the SI file depict the correlation between *ΔE*_*t*_ and *ΔE*_*t*_^*FD*^ and *ΔE*_*t*_^*DD*^ errors, respectively. Figure S4 shows an almost perfect correlation between *ΔE*_*t*_ and *ΔE*_*t*_^*FD*^, confirming the dominant role of FD error for all hybrid DFAs. We recall that the FD error depends on only ρ^*ref*^, thus showing solely the impact related to the analytical form of DFA. Figure S5, in turn, shows that DD errors produced by GKS hybrid realization are always 2–3 orders of magnitude smaller than FD counterparts. This is not surprising, since, in most cases, the electronic density enters the XC energy expressions nonlinearly, making it less sensitive to the choice of input density. This is depicted in Figure S6 in the SI file, where we correlate averaged *ΔE*_*t*_^*DD*^ and *Δρ* errors for a given hybrid DFA. One can note that the DD error is usually 1–2 orders smaller than *Δρ*. This most probably suggests that the FD errors in the functionals are transferred via the SCF procedure to the electron density visible in block B3.

The final block B4 is dominated by functionals (55 cases) for which *Δρ* < *Δρ*^HF^ and *ΔE*_*t*_ < *ΔE*_*t*_^HF^. In this region, we also note all tested *ab initio* post-HF methods, which are close to the “ideal” error-decreasing trend indicated by the diagonal dash-dotted line. Here, we can find, e.g., PBE0,^67^ r^2^SCAN0,^68^ CAM-QTP-00,^62^ or ωB97X-V^69^ DFAs. For total energy error, one can note that some DFAs (mostly GGA and RS-GGA hybrids) perform better than MP2 and usually do not exceed MP3 performance. The only exception is found for the smallest *ΔE*_*t*_ reported by three global hybrid GGAs, APF, MPW1PBE, and REVB3LYP, also shown in Table 2. In these cases, the accuracy is comparable to that of the MP3 results. In the same table, we also report the three smallest errors for *Δρ*, which are yielded by TPSS0,^70^ revTPSSH,^71^ and PBE50^72^ hybrid DFAs. Also, here the best hybrids do not exceed the MP2 performance. Additional inspection of Figures S4 and S5 in the SI file indicates that all functionals in the B4 segment of Figure 4 are characterized by small FD and DD errors. Because of that and noting small errors in electronic density, one can conclude that the functionals in the B4 block, due to various physical constraints built into the XC formula, provide accurate results for good reasons.

**Table 2:** Three Hybrid XC DFAs with the Lowest Average Errors Calculated for the CCSD(T) Benchmark Set

Error Type	XC Functional	Type	Error
Δ*E*_*t*_	APF	GGA	0.0003
	MPW1PBE	GGA	0.0004
	REVB3LYP	GGA	0.0004
	HF	*ab initio*	0.0065
	MP2	*ab initio*	0.0009
	MP3	*ab initio*	0.0003
	CCSD	*ab initio*	0.0001
			
Δρ	TPSS0	MGGA	0.0015
	REVTPSSH	MGGA	0.0015
	PBE50	GGA	0.0016
	HF	*ab initio*	0.0032
	MP2	*ab initio*	0.0009
	MP3	*ab initio*	0.0005
	CCSD	*ab initio*	0.0001
			
Δ*v*_*xc*_	HFLYP	GGA	0.0884
	LC-WPBEH-WHS	RS-GGA	0.1002
	WB97X	RS-GGA	0.1015
	HF	*ab initio*	0.0922
	MP2	*ab initio*	0.0401
	MP3	*ab initio*	0.0359
	CCSD	*ab initio*	0.0205
			
Δρ^*ref*^*v*_*xc*_	HFLYP	GGA	0.0285
	R2SCAN50	MGGA	0.0419
	PBE-2X	GGA	0.0497
	HF	*ab initio*	0.0173
	MP2	*ab initio*	0.0090
	MP3	*ab initio*	0.0053
	CCSD	*ab initio*	0.0019
			
Δ*v*_*c*_	HFLYP	GGA	1.2657
	LC-WPBEH-WHS	RS-GGA	1.5593
	WB97X	RS-GGA	1.6344
	HF	*ab initio*	1.0
	MP2	*ab initio*	0.4621
	MP3	*ab initio*	0.4435
	CCSD	*ab initio*	0.3736
			
Δ*IP*	CAM-QTP-00	RS-GGA	0.0301
	LC-WPBEH-WHS	RS-GGA	0.0343
	RCAM-B3LYP	RS-GGA	0.0382
	HF	*ab initio*	0.0614
	MP2	*ab initio*	0.0348
	MP3	*ab initio*	0.0247
	CCSD	*ab initio*	0.0000

Now we turn our attention to *Δv*_*xc*_, *Δρ*^*ref*^*v*_*xc*_, and *ΔIP* errors reported in Figure 3 to analyze the behavior of XC potentials and IPs generated by all hybrid functionals. As mentioned, the XC potential is a well-defined quantity entering the KS scheme. It is often overlooked in the DFA development process, but it tremendously impacts the quality of the orbitals and eigenvalues (e.g., first IP). One can note that *Δρ*^*ref*^*v*_*xc*_ error is generally 1–2 orders larger than density and total energy errors. The *Δv*_*xc*_ error, in turn, gives much larger values for most DFAs (3–4 orders larger than *Δρ* errors). This confirms the findings from ref (32), where authors observe similar behavior for a few of the most popular global hybrids. However, the present analysis further reveals that this difference is much smaller for long-range corrected hybrids. This is due to the large HF contribution in the XC functional, which, in turn, recovers the proper asymptotic behavior of *v*_*xc*_ and thus reduces the *Δv*_*xc*_ error for these species (see also Section 3 in the SI file). This is depicted in Figure 5 where we report the value of *Δv*_*xc*_ as a function of HF contribution. One can note that the error is relatively large for most global hybrids compared to long-range corrected DFAs and *ab initio* methods. The same feature also significantly impacts the quality of the first IP obtained from all hybrid DFAs. An evident direct quality dependence between the first IP and the XC potential exists, as seen in Figure 6. The best IPs are yielded by the CAM-QTP-00 functional (see Table 2), which was constructed to provide the smallest IP error,^62^ which agrees with our findings. That indicates the ultimate necessity in DFT calculations to care for the high quality of the XC potential to provide better IPs, which also results directly from the structure of the KS equations. This offers alternative confirmation of previous findings.^30,73−76^ From Figure 3, we can see that the region with lower *v*_*xc*_ and IP error starts from the 119 functional, and it is highly dominated by RS-GGA and RS-MGGA XC functionals. In general, those functionals have long-range HF coefficients equal to 1, which results in a correct −1/*r* behavior of the XC potential in the asymptotic tail. On the other hand, the results displayed in Table 1 show that all functionals with the largest *v*_*xc*_ errors are mostly yielded by short-range corrected hybrids for which the long-range HF coefficients equal zero. Thus, except for some individual cases, the RS hybrids better describe *v*_*xc*_ effectively and hence have a lower error for IPs.

**Figure 5:**
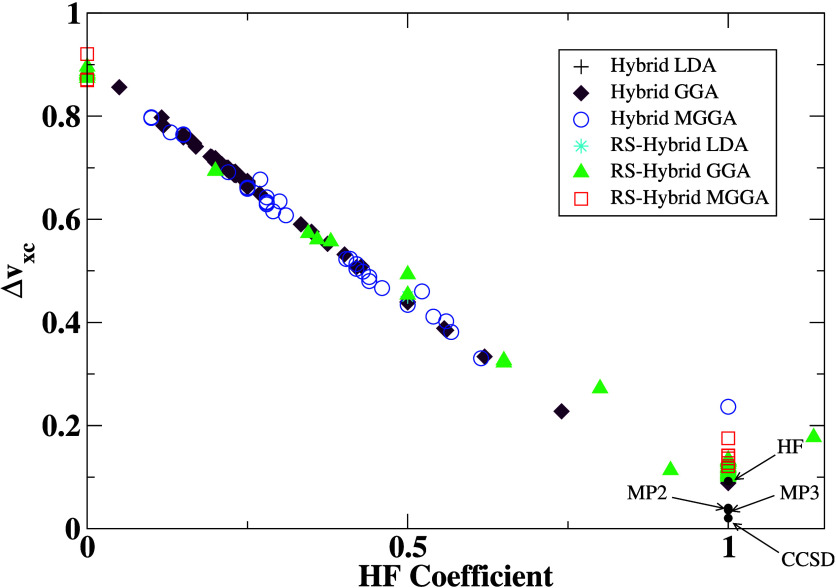
Average *Δv*_*xc*_ errors versus the HF coefficients (global hybrids) and long-range HF coefficients (range-separated hybrids).

**Figure 6:**
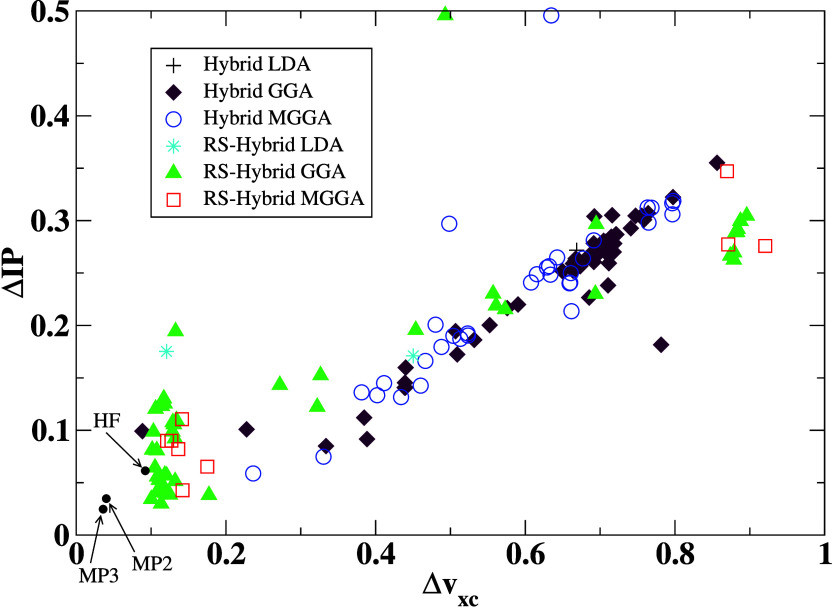
Average IP errors vs average errors of the exchange-correlation potential calculated for the CCSD(T) benchmark set. The IP EOM-CCSD method is used as a reference to calculate IP error.

The *Δρ*^*ref*^*v*_*xc*_ error, in turn, yields generally (for global and RS hybrids) an error varying between 0.02 and 1.29. This is presented in Figure S7 in the SI file on which we show the *Δρ*^*ref*^*v*_*xc*_ and density *Δρ* errors together with the *ab initio* results. As we see, the *Δρ*^*ref*^*v*_*xc*_ error is bigger for all hybrid functionals than the HF one and certainly MP2, MP3, and CCSD. Generally the *Δρ*^*ref*^*v*_*xc*_ error is notably smaller in almost all cases than the *Δv*_*xc*_ counterpart. This is an outcome of removing large contributions in the latter coming from the asymptotic tail observed for global hybrids. For this reason, *Δρ*^*ref*^*v*_*xc*_ error provides us more insight into the quality of the XC potential in the core and valence regions.

An inspection of Table 2 shows that the smallest *Δv*_*xc*_ error is provided by the HFLYP^77,78^ functional, which is a simple combination of 100% HF contribution and 100% of LYP^77,78^ correlation functional. It provides very good Δ*v*_*xc*_, Δ*ρ*^*ref*^*v*_*xc*_, and even the correlation-only potentials (Δ*v*_*c*_). This is closely followed by LC-WPBEH-WHS^79,80^ and WB97X,^81^ which yield similar errors. We note that the quality of the XC potentials can also be effectively examined by a direct visual comparison. This is done in Figures S8 and S9 (top row) in the SI file, where we report the XC potential obtained with HFLYP, LC-WPBEH-WHS, and CAM-QTP-00 DFAs for the Be atom and LiH molecule compared with FCI reference data. One can note that the hybrid potentials almost match the reference data in all cases. However, this can be misleading. Closer inspection of *Δv*_*xc*_ errors reveals that practically none of the existing hybrid DFAs can give a smaller error for *v*_*xc*_ than the one obtained for the HF method (*Δv*_*xc*_^*HF*^ = 0.0922). This is an astonishing result since HF density provides the exchange-only potential (without any correlation). This indicates that the correlation potential (*v*_*c*_) obtained for a given hybrid DFA possesses significant errors. In Table 2, we also report the smallest correlation potential error (*Δv*_*c*_). In this case, errors are larger than those of HF. This is confirmed by the inspection of correlation potentials depicted in Figures S8 and S9 (bottom row) of the SI file. The deviations are far more visible in this case, especially in the core and valence regions, which indicate that accurate *v*_*c*_ potentials are the main drawback of present day hybrid DFAs. Nevertheless, these *v*_*c*_ are very physical, being in phase with and having the shape of the reference FCI counterpart, which extends and confirms the finding from ref (30).

In conclusion, the study evaluates the quality of 155 hybrid functionals available in the LIBXC library with the main focus on their XC and correlation KS potentials, electron densities, ionization potentials, and total energies by direct comparison with the *ab initio* FCI and CCSD(T) reference data.

The analysis of total energies and electron densities revealed a group of approximations (mostly global hybrid GGAs and MGGAs) that mutually yield small errors for both quantities. Further analysis in terms of the DD and FD clearly indicates that the hybrid energy expression is mainly dominated by the FD error, which is related to the analytical form of DFA. Therefore, in order to find a better hybrid functional, one should focus on the maximal reduction of the FD error given by the analytical formula.

Because the XC potentials are not directly accessible for the DFT hybrids and *ab initio* references, we calculated them by inverting their electron densities. The first important finding from this analysis is that most global and RS hybrids produce similar shell structure oscillations in *v*_*xc*_ potentials that are comparable qualitatively with reference data. Further improvement can be seen for long-range corrected RS-GGA and RS-MGGA hybrids for which *v*_*xc*_ decays as −1/*r* in the asymptotic tail, providing the best overall performance of XC and correlation potentials. This explains the dramatic improvement upon global hybrids in the calculation of many properties.^82,83^ The same feature also impacts the quality of the first IPs, which benefit from the correct −1/*r* asymptotic behavior of *v*_*xc*_ incorporated by the HF long-range component. Increasing the contribution of the HF exchange term results in smaller errors for XC and IPs, for which we observe mutual direct and systematic dependence.

The closer inspection of *Δv*_*xc*_ and *Δv*_*c*_ errors reveals that practically none of the existing hybrid DFAs can give a smaller error than the one obtained for the HF method. This indicates that the correlation potential obtained for a given hybrid DFA possesses significant errors. Therefore, this finding emphasizes the ultimate need to inspect FD errors and XC and correlation KS potentials as a decisive criterion in constructing new and improved hybrid functionals.

We have provided the individual values of all averaged errors for all hybrid functionals via 10.18150/V4XZSF,^57^ which allows the reader to perform an additional assessment in the context of the applications and DFT functional development. It must be stressed here that hybrids with the highest errors should be avoided in actual DFT applications. We think that the present analysis can help overcome current limitations and face essential challenges in constructing new XC DFAs within the KS-DFT framework.

## Data Availability

The data that support the findings are published within this study.

## Supporting Information Available

The table with the list of all hybrid functionals, additional figures showing error correlations, figures with XC, and correlation potentials (PDF)
Transparent Peer Review report available (PDF)
