# Supplementary material for: A Critical Evaluation of the Hybrid KS DFT Functionals Based on the KS Exchange-Correlation Potentials
Source: J Phys Chem Lett. 2024 Oct 2;15(40):10219–29. doi: 10.1021/acs.jpclett.4c01979 (PMC11472381; doi:10.1021/acs.jpclett.4c01979)
Supplement: Supplementary file 1 — jz4c01979_si_001.pdf [file jz4c01979_si_001.pdf]

Supplementary Materials for "A Critical Evaluation of the Hybrid KS DFT Functionals Based on the KS Exchange-Correlation Potentials"

Vignesh Kumar, Szymon Śmiga*, Ireneusz Grabowski

Institute of Physics, Faculty of Physics, Astronomy, and Informatics, Nicolaus Copernicus University in Toruń,
ul. Grudziadzka 5, 87-100 Toruń, Poland

1 List of hybrid functionals

Table ST1: The list of 155 hybrid functionals used in the assessment. They are numbered according to the order described in the main manuscript and used in the figures. The names of the functionals are taken from the LIBXC library.

S.No	Hybrid Functional	Functional Type
1	HYB_LDA_XC_LDA0	Hybrid-LDA
2	HYB_GGA_XC_MPWLYP1M	Hybrid-GGA
3	HYB_GGA_XC_O3LYP	Hybrid-GGA
4	HYB_GGA_XC_WP04	Hybrid-GGA
5	HYB_GGA_XC_B97_1P	Hybrid-GGA
6	HYB_GGA_XC_B3LYPS	Hybrid-GGA
7	HYB_GGA_XC_B1WC	Hybrid-GGA
8	HYB_GGA_XC_HPBEINT	Hybrid-GGA
9	HYB_GGA_XC_EDF2	Hybrid-GGA
10	HYB_GGA_XC_SB98_1C	Hybrid-GGA
11	HYB_GGA_XC_B97	Hybrid-GGA
12	HYB_GGA_XC_B3LYP_MCM1	Hybrid-GGA
13	HYB_GGA_XC_SB98_1B	Hybrid-GGA
14	HYB_GGA_XC_REVB3LYP	Hybrid-GGA
15	HYB_GGA_XC_B3PW91	Hybrid-GGA
16	HYB_GGA_XC_MPW3PW	Hybrid-GGA
17	HYB_GGA_XC_B3P86_NWCHEM	Hybrid-GGA
18	HYB_GGA_XC_B3P86	Hybrid-GGA
19	HYB_GGA_XC_MB3LYP_RC04	Hybrid-GGA
20	HYB_GGA_XC_B3LYP	Hybrid-GGA

Continuation of Table S1

S.No	Hybrid Functional	Functional Type
21	HYB_GGA_XC_B3LYP3	Hybrid-GGA
22	HYB_GGA_XC_B3LYP5	Hybrid-GGA
23	HYB_GGA_XC_HAPBE	Hybrid-GGA
24	HYB_GGA_XC_B97_2	Hybrid-GGA
25	HYB_GGA_XC_B97_1	Hybrid-GGA
26	HYB_GGA_XC_X3LYP	Hybrid-GGA
27	HYB_GGA_XC_MPW3LYP	Hybrid-GGA
28	HYB_GGA_XC_SB98_2C	Hybrid-GGA
29	HYB_GGA_XC_B3LYP_MCM2	Hybrid-GGA
30	HYB_GGA_XC_SB98_1A	Hybrid-GGA
31	HYB_GGA_XC_APF	Hybrid-GGA
32	HYB_GGA_XC_RELPBE0	Hybrid-GGA
33	HYB_GGA_XC_SB98_2A	Hybrid-GGA
34	HYB_GGA_XC_SB98_2B	Hybrid-GGA
35	HYB_GGA_XC_CAP0	Hybrid-GGA
36	HYB_GGA_XC_PBE_SOL0	Hybrid-GGA
37	HYB_GGA_XC_PBEH	Hybrid-GGA
38	HYB_GGA_XC_PBEB0	Hybrid-GGA
39	HYB_GGA_XC_CASE21	Hybrid-GGA
40	HYB_GGA_XC_MPW1PBE	Hybrid-GGA
41	HYB_GGA_XC_B1PW91	Hybrid-GGA
42	HYB_GGA_XC_MPW1PW	Hybrid-GGA
43	HYB_GGA_XC_B1LYP	Hybrid-GGA
44	HYB_GGA_XC_MPW1LYP	Hybrid-GGA
45	HYB_GGA_XC_APBE0	Hybrid-GGA
46	HYB_GGA_XC_PBE_MOL0	Hybrid-GGA
47	HYB_GGA_XC_PBE_MOLB0	Hybrid-GGA
48	HYB_GGA_XC_B97_3	Hybrid-GGA
49	HYB_GGA_XC_PBE0_13	Hybrid-GGA
50	HYB_GGA_XC_BLYP35	Hybrid-GGA
51	HYB_GGA_XC_PBE38	Hybrid-GGA
52	HYB_GGA_X_SOGGA11_X,GGA_C_SOGGA11_X	Hybrid-GGA
53	HYB_GGA_XC_B97_K	Hybrid-GGA
54	HYB_GGA_XC_MPW1K	Hybrid-GGA
55	HYB_GGA_XC_PBE50	Hybrid-GGA
56	HYB_GGA_XC_B5050LYP	Hybrid-GGA
57	HYB_GGA_XC_BHANDHLYP	Hybrid-GGA
58	HYB_GGA_XC_BHANDH	Hybrid-GGA
59	HYB_GGA_XC_KMLYP	Hybrid-GGA
60	HYB_GGA_XC_PBE_2X	Hybrid-GGA

Continuation of Table S1

S.No	Hybrid Functional	Functional Type
61	HYB_GGA_XC_QTP17	Hybrid-GGA
62	HYB_GGA_XC_WC04	Hybrid-GGA
63	HYB_GGA_XC_HFLYP	Hybrid-GGA
64	HYB_MGGA_XC_R2SCANH	Hybrid-MGGA
65	HYB_MGGA_XC_TPSSH	Hybrid-MGGA
66	HYB_MGGA_XC_REVTPSSH	Hybrid-MGGA
67	HYB_MGGA_XC_TPSS1KCIS	Hybrid-MGGA
68	HYB_MGGA_X_TAU_HCTH,GGA_C_HYB_TAU_HCTH	Hybrid-MGGA
69	HYB_MGGA_XC_MPW1KCIS	Hybrid-MGGA
70	HYB_MGGA_XC_PBE1KCIS	Hybrid-MGGA
71	HYB_MGGA_X_SCAN0,MGGA_C_SCAN	Hybrid-MGGA
72	HYB_MGGA_XC_R2SCAN0	Hybrid-MGGA
73	HYB_MGGA_XC_TPSS0	Hybrid-MGGA
74	HYB_MGGA_XC_B0KCIS	Hybrid-MGGA
75	HYB_MGGA_X_M06,MGGA_C_M06	Hybrid-MGGA
76	HYB_MGGA_XC_B88B95	Hybrid-MGGA
77	HYB_MGGA_XC_B86B95	Hybrid-MGGA
78	HYB_MGGA_XC_PW6B95	Hybrid-MGGA
79	HYB_MGGA_X_M05,MGGA_C_M05	Hybrid-MGGA
80	HYB_MGGA_XC_PW86B95	Hybrid-MGGA
81	HYB_MGGA_XC_X1B95	Hybrid-MGGA
82	HYB_MGGA_XC_MPW1B95	Hybrid-MGGA
83	HYB_MGGA_X_REVM06,MGGA_C_REVM06	Hybrid-MGGA
84	HYB_MGGA_XC_MPWKCIS1K	Hybrid-MGGA
85	HYB_MGGA_XC_BB1K	Hybrid-MGGA
86	HYB_MGGA_X_BMK,GGA_C_BMK	Hybrid-MGGA
87	HYB_MGGA_XC_XB1K	Hybrid-MGGA
88	HYB_MGGA_XC_MPWB1K	Hybrid-MGGA
89	HYB_MGGA_X_MN15,MGGA_C_MN15	Hybrid-MGGA
90	HYB_MGGA_XC_PWB6K	Hybrid-MGGA
91	HYB_MGGA_XC_R2SCAN50	Hybrid-MGGA
92	HYB_MGGA_X_M08_HX,MGGA_C_M08_HX	Hybrid-MGGA
93	HYB_MGGA_X_M06_2X,MGGA_C_M06_2X	Hybrid-MGGA
94	HYB_MGGA_X_M08_SO,MGGA_C_M08_SO	Hybrid-MGGA
95	HYB_MGGA_X_DLDF,MGGA_C_DLDF	Hybrid-MGGA
96	HYB_MGGA_X_M05_2X,MGGA_C_M05_2X	Hybrid-MGGA
97	HYB_MGGA_X_M06_HF,MGGA_C_M06_HF	Hybrid-MGGA
98	HYB_LDA_XC_CAM_LDA0	RS-Hybrid-LDA
99	HYB_LDA_XC_BN05	RS-Hybrid-LDA
100	HYB_GGA_XC_HSE12S	RS-Hybrid-GGA

Continuation of Table S1

S.No	Hybrid Functional	Functional Type
101	HYB_GGA_XC_HSE_SOL	RS-Hybrid-GGA
102	HYB_GGA_XC_HJS_PBE_SOL	RS-Hybrid-GGA
103	HYB_GGA_XC_HSE12	RS-Hybrid-GGA
104	HYB_GGA_XC_HJS_B97X	RS-Hybrid-GGA
105	HYB_GGA_XC_HJS_PBE	RS-Hybrid-GGA
106	HYB_GGA_XC_HSE03	RS-Hybrid-GGA
107	HYB_GGA_XC_HSE06	RS-Hybrid-GGA
108	HYB_GGA_X_N12_SX,GGA_C_N12_SX	RS-Hybrid-GGA
109	HYB_GGA_XC_CAM_PBEH	RS-Hybrid-GGA
110	HYB_GGA_XC_CAMY_PBEH	RS-Hybrid-GGA
111	HYB_GGA_X_CAM_S12G,GGA_C_PBE	RS-Hybrid-GGA
112	HYB_GGA_X_CAM_S12H,GGA_C_PBE	RS-Hybrid-GGA
113	HYB_GGA_XC_MCAM_B3LYP	RS-Hybrid-GGA
114	HYB_GGA_XC_CAMH_B3LYP	RS-Hybrid-GGA
115	HYB_GGA_XC_WHPBE0	RS-Hybrid-GGA
116	HYB_GGA_XC_CAM_B3LYP	RS-Hybrid-GGA
117	HYB_GGA_XC_CAMY_B3LYP	RS-Hybrid-GGA
118	HYB_GGA_XC_CAM_O3LYP	RS-Hybrid-GGA
119	HYB_GGA_XC_CAM_QTP_00	RS-Hybrid-GGA
120	HYB_GGA_XC_LC_WPBEH_WHS	RS-Hybrid-GGA
121	HYB_GGA_XC_WB97X_D	RS-Hybrid-GGA
122	HYB_GGA_XC_LC_WPBESOL_WHS	RS-Hybrid-GGA
123	HYB_GGA_XC_CAM_QTP_02	RS-Hybrid-GGA
124	HYB_GGA_XC_LRC_WPBEH	RS-Hybrid-GGA
125	HYB_GGA_XC_WB97X_V	RS-Hybrid-GGA
126	HYB_GGA_XC_WB97X_D3	RS-Hybrid-GGA
127	HYB_GGA_XC_CAM_QTP_01	RS-Hybrid-GGA
128	HYB_GGA_XC_CAMY_BLYP	RS-Hybrid-GGA
129	HYB_GGA_XC_WB97X	RS-Hybrid-GGA
130	HYB_GGA_XC_LCY_PBE	RS-Hybrid-GGA
131	HYB_GGA_XC_LC_VV10	RS-Hybrid-GGA
132	HYB_GGA_XC_LC_WPBE08_WHS	RS-Hybrid-GGA
133	HYB_GGA_XC_LC_WPBE_WHS	RS-Hybrid-GGA
134	HYB_GGA_XC_LRC_WPBE	RS-Hybrid-GGA
135	HYB_GGA_XC_TUNED_CAM_B3LYP	RS-Hybrid-GGA
136	HYB_GGA_XC_LC_PBEOP	RS-Hybrid-GGA
137	HYB_GGA_XC_LC_BOP	RS-Hybrid-GGA
138	HYB_GGA_XC_LC_WPBE	RS-Hybrid-GGA
139	HYB_GGA_XC_LC_QTP	RS-Hybrid-GGA
140	HYB_GGA_XC_WB97	RS-Hybrid-GGA

Continuation of Table S1

141	HYB_GGA_XC_LCY_BLYP	RS-Hybrid-GGA
142	HYB_GGA_XC_LC_BLYP	RS-Hybrid-GGA
143	HYB_GGA_XC_LC_BLYPR	RS-Hybrid-GGA
144	HYB_GGA_XC_LC_BLYP_EA	RS-Hybrid-GGA
145	HYB_GGA_XC_LB07	RS-Hybrid-GGA
146	HYB_GGA_XC_RCAM_B3LYP	RS-Hybrid-GGA
147	HYB_MGGA_X_JS18,MGGA_C_TPSS	RS-Hybrid-MGGA
148	HYB_MGGA_X_MN12_SX,MGGA_C_MN12_SX	RS-Hybrid-MGGA
149	HYB_MGGA_X_M06_SX,MGGA_C_M06_SX	RS-Hybrid-MGGA
150	HYB_MGGA_XC_WB97M_V	RS-Hybrid-MGGA
151	HYB_MGGA_X_PJS18,GGA_C_LYP	RS-Hybrid-MGGA
152	HYB_MGGA_XC_LC_TMLYP	RS-Hybrid-MGGA
153	HYB_MGGA_X_M11,MGGA_C_M11	RS-Hybrid-MGGA
154	HYB_MGGA_X_REVM11,MGGA_C_REVM11	RS-Hybrid-MGGA
155	HYB_MGGA_XC_GAS22	RS-Hybrid-MGGA

2 Computational details

2.1 Wu-Yang method

We employ the Wu-Yang (WY) inverse Kohn-Sham (KS) method [5, 4, 3] to compute the KS exchange-correlation (XC) potential corresponding to a given input density. The input densities (ρ_{in}) are obtained from the *ab initio* FCI, CCSD(T), MP3, MP2, HF methods, and the hybrid GKS calculations. The inverse method is based on the unconstrained maximization of the WY functional $W_s[\psi_{det}, v(\boldsymbol{r})]$ which is defined as

$$W_s[\psi_{det}, v(\boldsymbol{r})] = 2\sum_{i}^{N/2} \langle\phi_i| \hat{T} |\phi_i\rangle + \int d\boldsymbol{r} v_s(\boldsymbol{r})[\rho(\boldsymbol{r}) - \rho_{in}(\boldsymbol{r})], \tag{1}$$

where ψ_{det} is the system's wavefunction, and ϕ_i are the KS orbitals. v_s is the effective KS potential constructed as

$$v_s(\boldsymbol{r}) = v_{ext}(\boldsymbol{r}) + v_0(\boldsymbol{r}) + v_{pbs}(\boldsymbol{r}), \tag{2}$$

where v_{ext} is the external potential, v_0 is the guide potential usually fixed as the Fermi-Amaldi potential, and v_{pbs} is the rest of the potential expanded in the auxiliary finite potential basis set. Once $v_s(\boldsymbol{r})$ is obtained, the XC potential can be computed in a straightforward way, such as

$$v_{xc}(\boldsymbol{r}) = v_0(\boldsymbol{r}) + v_{pbs}(\boldsymbol{r}) - v_h(\boldsymbol{r}) \tag{3}$$

where v_h is the Hartree potential.

In our calculations, the FCI and CCSD(T) reference electron densities are obtained using the Psi4 [6] routines. Their corresponding reference XC potentials, in turn, are calculated via the WY scheme using the *n2v* package with Psi4[6, 7] as the engine. The hybrid XC potentials are computed using the same WY inversion with the *n2v* and PySCF[8, 10] program package. In all the WY calculations, the lambda regularization parameter[4], which prevents nonphysical v_{xc} oscillations near the core, is set to 10^{-5}. In addition, the Trust-Exact optimization method with a convergence tolerance of 10^{-6} and the Fermi-Amaldi guide potential is employed to correct the asymptotic behavior of the XC potentials.

2.2 Grid and basis set convergence

To ensure that our results do not depend on the DFT grid and basis set used in the calculations, we have performed an extended analysis of such dependence. In our calculations, we use the DFT grid described by the Lebedev-Laiko quadrature formula as implemented in the PySCF package. The standard PySCF calculations used a predefined grid corresponding to level 5. To check how the results depend on the choice of the DFT grid, we also perform calculations using a larger DFT grid constructed with 131 radial and 4334 angular grid points (He_2 molecule). In Figure S1, we present the convergence of various errors concerning DFT grid size calculated for the He_2 molecule with the FCI reference. The qualitative behavior of all errors is almost identical. Thus, we conclude that the errors are well-converged w.r.t. grid size. A similar test was performed to check the basis set convergence of all errors. This is reported in Figure S2 for the case of He_2 molecule with the FCI reference, using an uncontracted aug-cc-pVTZ basis set [2] and a much larger uncontracted aug-cc-pV5Z basis set [1]. Also, here, the qualitative behavior of all errors is very similar despite the size of the basis set used in the calculations.

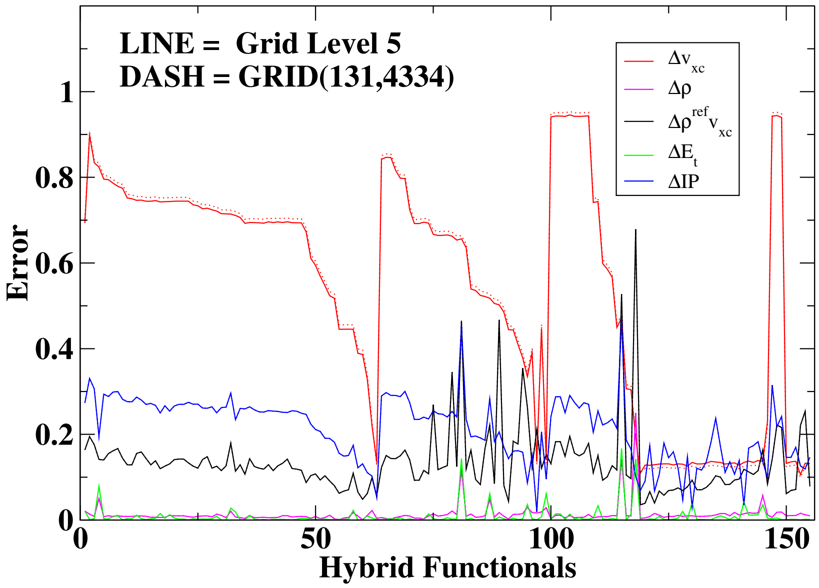

Figure S1: Errors calculated for two different DFT grid levels, 5 (Line) and (131,4334) (Dash), for He_2 and FCI reference. The IP-EOM-CCSD method is used as a reference to calculate IP error.

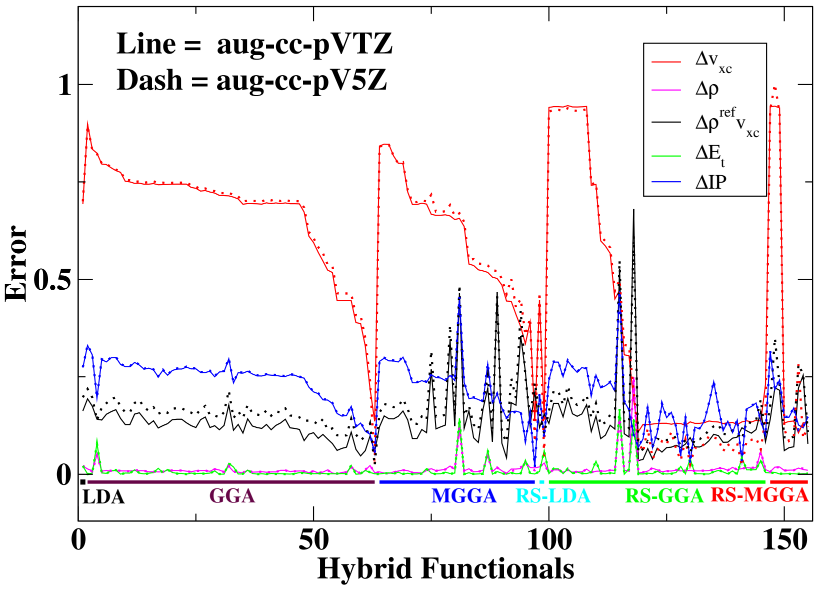

Figure S2: Errors calculated for He_2 molecule with FCI reference, using two different basis sets: uncontracted aug-cc-pVTZ (Line) and uncontracted aug-cc-pV5Z (Dash). The IP-EOM-CCSD method is used as a reference to calculate IP error.

3 The asymptotic behavior of Δv_{xc} error

Let us consider an atom with the reference v_{xc}^{ref} potential generated from the WY method. When $r \to \infty$, it behaves as $v_{xc}^{ref} \approx -\frac{1}{r} + C$ where C is a constant which emerges from the fact that XC WY potential can be computed up to a constant. On the other hand, the hybrid asymptotic behavior is governed by mutual semilocal and Hartree-Fock exchange contributions in the full XC energy expression as discussed in Ref. [9]. This could be in general written as $v_{xc} \approx -\frac{\alpha}{r} + (1-\alpha)f(r) + C'$ where $f(r)$ denotes the asymptotic decay of the semilocal exchange potential (e.g., $f(r) = -\frac{1}{r^2}$ and $f(r) = e^{-r}$, for B88 and PBE exchange, respectively) and C' is the constant. Thus, the asymptotic behavior of $\delta v_{xc} = v_{xc}^{ref} - v_{xc}$ error is given by

$$\delta v_{xc} \approx \frac{\alpha - 1}{r} - (1-\alpha)f(r) + C - C' \ . \tag{4}$$

If $\alpha = 1$ (100% HF contribution in the hybrid functional), then $\delta v_{xc} \approx C - C'$. Thus, a small (non-zero) systematic error still contributes to all integration grid points. This indicates, in turn, that δv_{xc} can take zero value only for the case when $v_{xc} = v_{xc}^{ref}$. On the other hand, if $\alpha = 0$, we have $\delta v_{xc} \approx -\frac{1}{r} - f(r) + C - C'$. Therefore, the Δv_{xc} error has a significant contribution from the asymptotic region, which governs the behavior of the error. This is also the case for all intermediate values of $\alpha \in < 0, 1 >$.

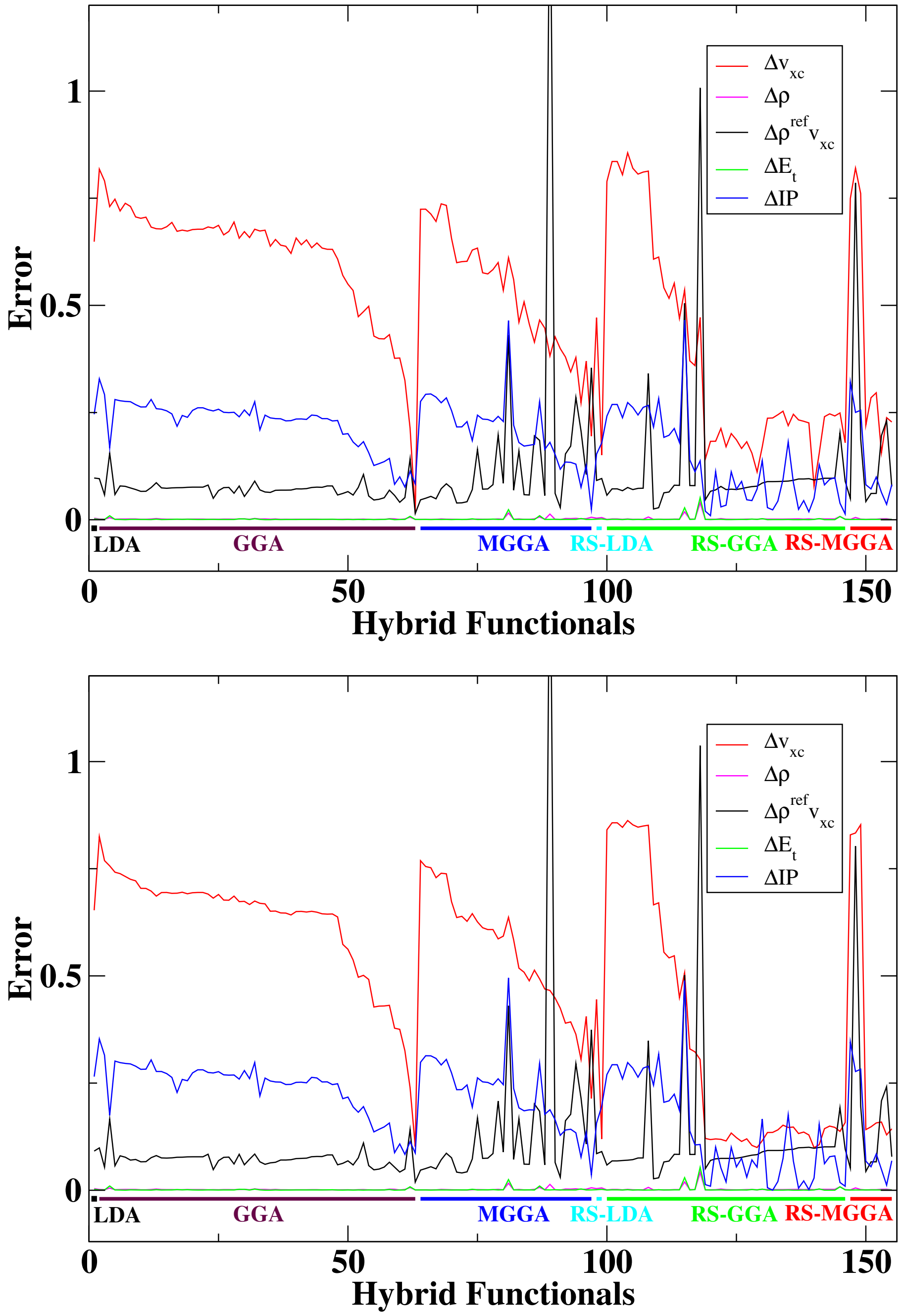

Figure S3: Errors defined by Eqs.(2)-(6) in the main manuscript calculated for Ar (top) atom and HCl (bottom) molecule with the CCSD(T) reference. The IP-EOM-CCSD method is used as a reference to calculate IP error. The X-axis corresponds to various hybrid functionals numbered and grouped according to the Tab. ST1.

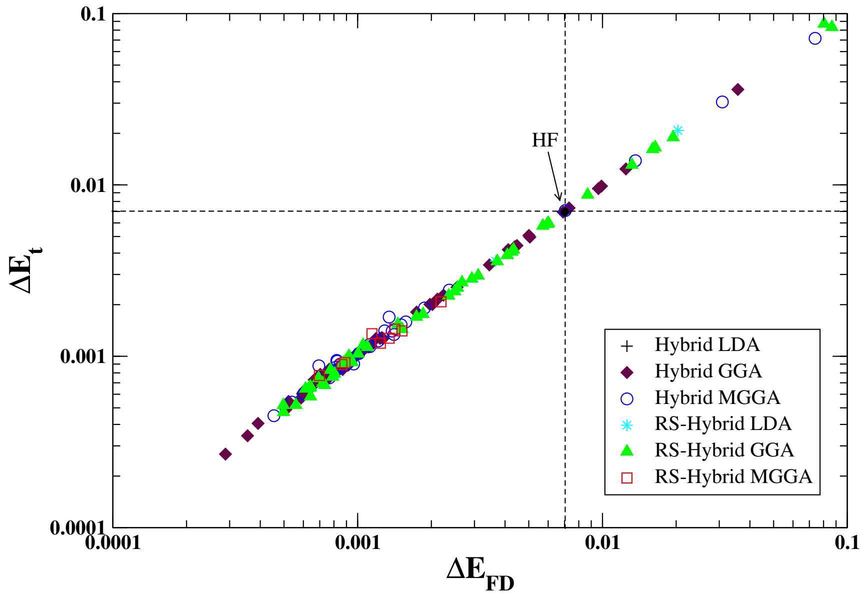

Figure S4: Average total energy error (ΔE_t) vs average functional-driven error (ΔE_{FD}) obtained for the CCSD(T) benchmark set. In both axes, the logarithmic scale is used.

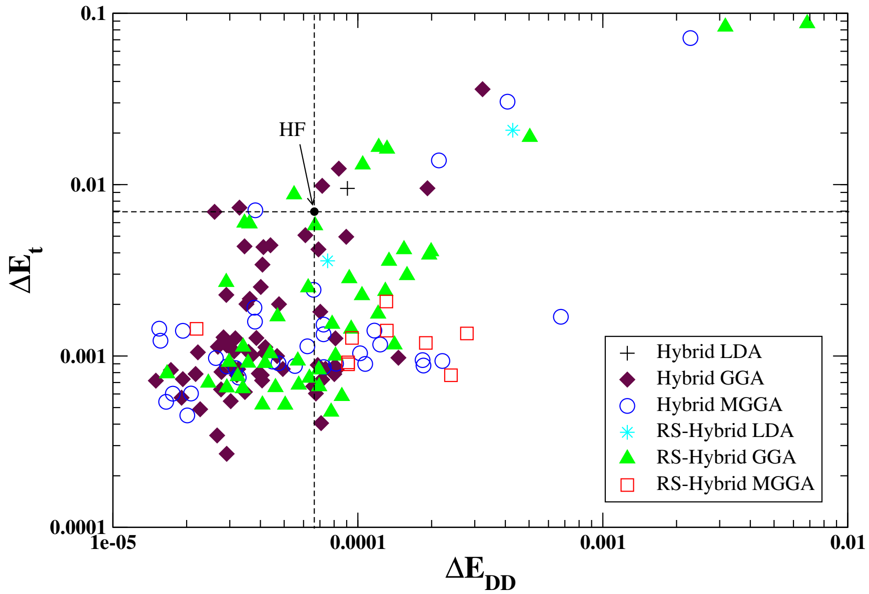

Figure S5: Average total energy error (ΔE_t) vs average density driven error (ΔE_{DD}) calculated for the CCSD(T) benchmark set. In both axes, the logarithmic scale is used.

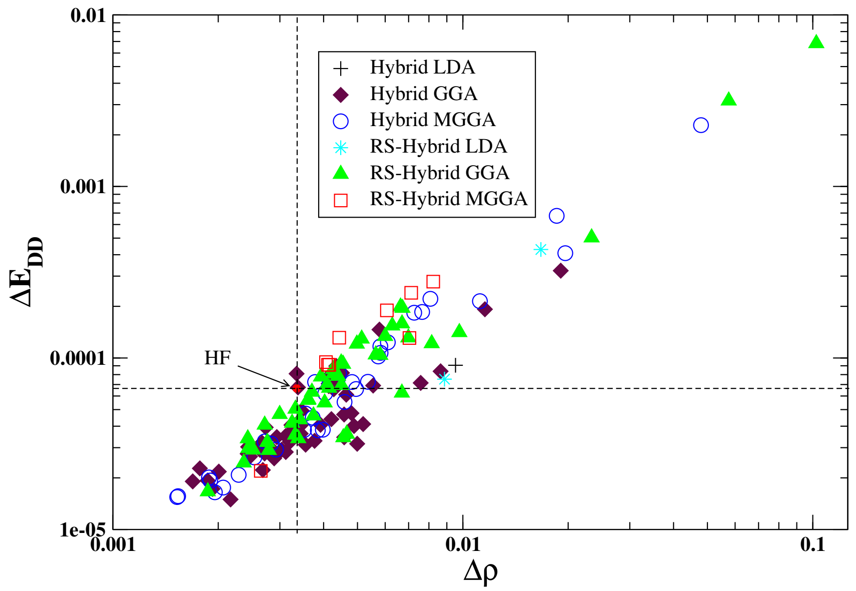

Figure S6: Average density-driven error (ΔE_{DD}) vs an average density error ($\Delta \rho$) obtained for CCSD(T) benchmark set. In both axes, the logarithmic scale is used.

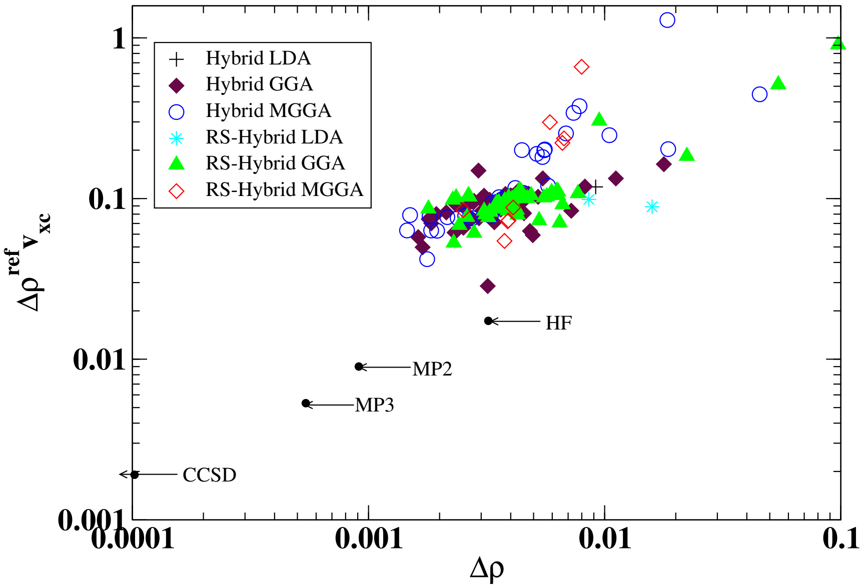

Figure S7: Average $\Delta \rho^{ref} v_{xc}$ error vs average density $\Delta \rho$ error calculated for the CCSD(T) benchmark set. In both axes, the logarithmic scale is used.

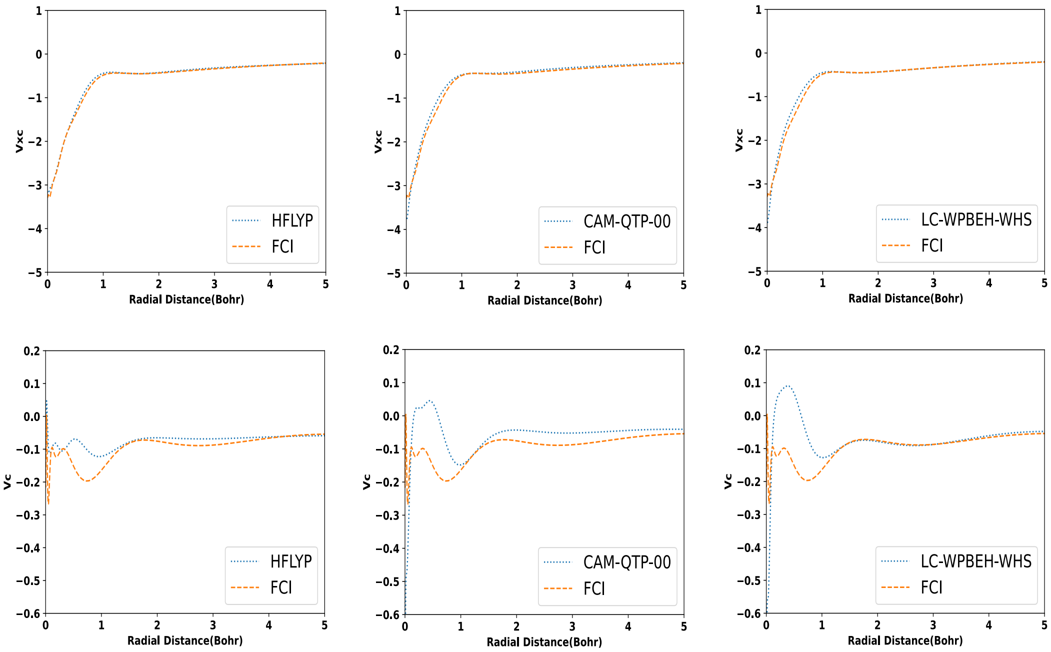

Figure S8: Exchange-Correlation potentials (Top Row) and correlation potentials (Bottom Row) computed using the Wu-Yang inverse method for Be atom for different functionals. The reference potentials are obtained from the FCI density.

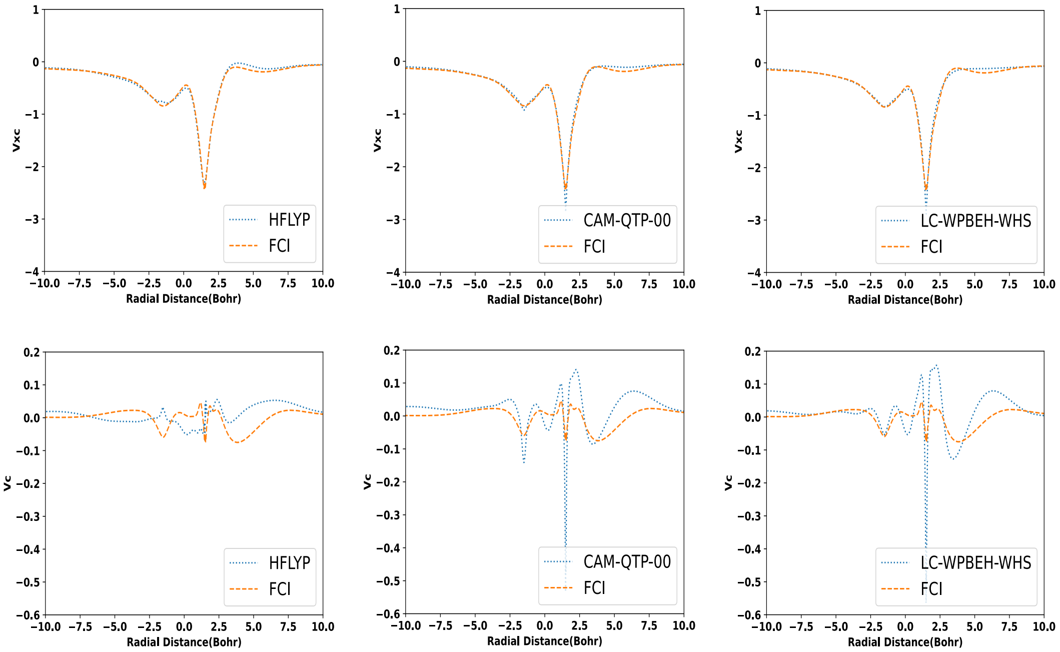

Figure S9: Exchange-Correlation potentials (Top Row) and correlation potentials (Bottom Row) computed using the Wu-Yang inverse procedure for LiH molecule for different functionals. The reference potentials are obtained from the FCI density.

References

[1] Jr. Dunning Thom H. "Gaussian basis sets for use in correlated molecular calculations. I. The atoms boron through neon and hydrogen". In: *The Journal of Chemical Physics* 90.2 (Jan. 1989), pp. 1007–1023. ISSN: 0021-9606. DOI: 10.1063/1.456153. eprint: https://pubs.aip.org/aip/jcp/article-pdf/90/2/1007/18974738/1007_1_online.pdf. URL: https://doi.org/10.1063/1.456153.

[2] Jr. David E. Woon; Thom H. Dunning. "Gaussian basis sets for use in correlated molecular calculations. III. The atoms aluminum through argon". In: *J. Chem. Phys* 98.2 (1993), pp. 1358–1371. DOI: 10.1063/1.464303.

[3] Qin Wu and Weitao Yang. "A direct optimization method for calculating density functionals and exchange–correlation potentials from electron densities". In: *The Journal of Chemical Physics* 118.6 (Jan. 2003), pp. 2498–2509. ISSN: 0021-9606. DOI: 10.1063/1.1535422. eprint: https://pubs.aip.org/aip/jcp/article-pdf/118/6/2498/10849003/2498_1_online.pdf. URL: https://doi.org/10.1063/1.1535422.

[4] Felipe A. Bulat et al. "Optimized effective potentials from electron densities in finite basis sets". In: *The Journal of Chemical Physics* 127.17 (2007), p. 174101. DOI: 10.1063/1.2800021. eprint: https://doi.org/10.1063/1.2800021. URL: https://doi.org/10.1063/1.2800021.

[5] Tim Heaton-Burgess, Felipe A. Bulat, and Weitao Yang. "Optimized Effective Potentials in Finite Basis Sets". In: *Phys. Rev. Lett.* 98 (25 June 2007), p. 256401. DOI: 10.1103/PhysRevLett.98.256401. URL: https://link.aps.org/doi/10.1103/PhysRevLett.98.256401.

[6] Robert M. Parrish et al. "Psi4 1.1: An Open-Source Electronic Structure Program Emphasizing Automation, Advanced Libraries, and Interoperability". In: *Journal of Chemical Theory and Computation* 13.7 (2017). PMID: 28489372, pp. 3185–3197. DOI: 10.1021/acs.jctc.7b00174. eprint: https://doi.org/10.1021/acs.jctc.7b00174. URL: https://doi.org/10.1021/acs.jctc.7b00174.

[7] Daniel G. A. Smith et al. "Psi4NumPy: An Interactive Quantum Chemistry Programming Environment for Reference Implementations and Rapid Development". In: *Journal of Chemical Theory and Computation* 14.7 (2018). PMID: 29771539, pp. 3504–3511. DOI: 10.1021/acs.jctc.8b00286. eprint: https://doi.org/10.1021/acs.jctc.8b00286. URL: https://doi.org/10.1021/acs.jctc.8b00286.

[8] Qiming Sun et al. "PySCF: the Python-based simulations of chemistry framework". In: *WIREs Computational Molecular Science* 8.1 (2018), e1340. DOI: https://doi.org/10.1002/wcms.1340. eprint: https://wires.onlinelibrary.wiley.com/doi/pdf/10.1002/wcms.1340. URL: https://wires.onlinelibrary.wiley.com/doi/abs/10.1002/wcms.1340.

[9] Szymon Śmiga and Lucian A. Constantin. "Unveiling the Physics Behind Hybrid Functionals". In: *J. Phys. Chem. A* 124.27 (2020), pp. 5606–5614.

[10] Qiming Sun et al. "Recent developments in the PySCF program package". In: *The Journal of Chemical Physics* 153.2 (2020), p. 024109. DOI: 10.1063/5.0006074. eprint: https://doi.org/10.1063/5.0006074. URL: https://doi.org/10.1063/5.0006074.
