# Supplementary material for: A Critical Evaluation of the Hybrid KS DFT Functionals Based on the KS Exchange-Correlation Potentials
Source: J Phys Chem Lett. 2024 Oct 2;15(40):10219–29. doi: 10.1021/acs.jpclett.4c01979 (PMC11472381; doi:10.1021/acs.jpclett.4c01979)
Supplement: Supplementary file 2 — jz4c01979_si_002.pdf [file jz4c01979_si_002.pdf]

jz-2024-019793.R1

Name: Peer Review Information for "A critical evaluation of the Hybrid KS DFT Functionals based on the KS exchange-correlation potentials"

First Round of Reviewer Comments

Reviewer: 1

Comments to the Author

Śmiga and co-workers proposed a new method for evaluating the quality of hybrid functionals. The procedure is novel and well-developed, which can significantly improve the future development of hybrid functionals. The method is described in detail, and the performed calculations are adequate, so I have only a few technical comments, primarily related to data presentation and evaluation. After clarifying these points, I would recommend the manuscript for publication in JPC Letters.

Comments:

1. It is not advisable to plot quantities with significantly different error magnitudes on the same graph. In Fig. 1-3, the errors for \Delta E_t and \Delta \rho are lost among other quantities. While this representation can show which quantities have more dominant relative errors, it might make interpreting the correlations a bit difficult. More comprehensible figures would be more valuable.

2. It is commendable that the authors separately examined the correctness of the XC potential in the core and valence regions. It would be important to mention for which properties the accurate description of these regions is crucial (e.g., atomization energies) and for which properties it is less critical (e.g., long-range interactions).

3. If possible, the authors could compare the performance of the best functionals with the results obtained for the GMTKN55 benchmark set if those are available. It would be interesting to see how the results for various physical and chemical properties correlate with the results discussed here (see the previous question).

4. I am interested in the authors' opinion on the following sentence: "To summarise our main findings in one sentence, we can state that we presented clear evidence that the vast majority of non-empirical DHDFs do not live up to the initial hopes that some in the developer community may have had [doi: 10.1039/c8cp03852j]." The authors' opinion in the cited manuscript is that only the final numerical results matter. It might be worthwhile to discuss this thought in detail in the present manuscript, considering whether the authors agree or disagree with this opinion.

5. The authors examined the obtained results depending on the grid. It might be interesting to see whether using different quadratures, such as Mura–Knowles, Gauss–Chebyshev, Treutler–Ahlrichs, and Euler–Maclaurin, affects the results. I guess that it does not influence the results too much, but I would be interested to know if the authors have any experience with this.

6. The procedure can be easily generalized for double hybrid functionals as well if I understand correctly. Due to local MP2 implementations, the use of these functionals may become increasingly dominant in the community. Do the authors plan to investigate these functionals as well?

Reviewer: 2

Comments to the Author

Kumar and coworkers develop a novel methodology to assess the accuracy of hybrid density-functional approximations in modeling some fundamental energy and density properties. Their methodology is somehow original since it focuses on error measurements calculated via the exchange-correlation potential of molecular systems while more conventional benchmarks are used to compare energy differences for a given property. In this sense, we should acknowledge the novelty of their work. With their methodology, they assess the accuracy of a very large panel of hybrid functionals which is by far representative of the DFT functional landscape.

Despite these novelties, I am not convinced that this work is suitable for the large and broad audience of JPCL. The research topic is here very specific toward developers of density-functional approximations, and to me, would be more suitable for a possible publication in JCTC or JPCA for the following reasons:

1 - molecules of interest in this work are very small and thus without chemical significance in comparison to the ones belonging for instance to the GMTKN55 dataset.

2 - Figures 2 and 3 are hard to analyze for specialists. We can imagine the difficulty for non specialists. A graph function of the fraction exact-like exchange would be much more explicit, as well as focusing on the performance of commonly used hybr

ids in literature.

Regarding the methodology:

3 - it is not clear to me if the error measurements are computed via reference densities or self-consistent densities. Usually, errors are derived from self-consistent computations. What is here the incidence?

Regarding the bibliography:

4 - it is a pity that the authors did not compare their approach with existing benchmarks focusing on energy and density errors.

5 - Typos:

p.5 Hohengerg-Kohn

Author's Response to Peer Review Comments:

Author’s Response to Reviews of

A comprehensive assessment of hybrid KS DFT functionals

Vignesh Kumar, Szymon Smiga and Ireneusz Grabowski´

The Journal of Physical Chemistry Letters

Manuscript ID: jz-2024-019793

RC: *Reviewer’s Comment*, AR: *Author’s Response*

Dear Editor,

thank you for your communication on August 7th, 2024. Please find our response to the Referee’s reports and a summary of the changes made to the manuscript. In the revised manuscript, we considered all the suggestions pointed out by the Referees.

We hope the manuscript may find your approval for publication. We look forward to this work’s publication in The Journal of Physical Chemistry Letters, ACS.

Sincerely,
Szymon Smiga (for all the authors)´

We thank both Referees for reviewing our manuscript and for valuable suggestions and comments. We respond point by point to the Referees's comments. Changes done in the present form of the manuscript are indicated in the color format.

Major changes in the manuscript

- We have amended the title as suggested by the editor
- We have added figure (Fig 3 (Bottom Panel)) where we present errors of total energy and density
- We have added a few sentences to address a comment from the reviewers

Reviewer #1

RC: 1. It is not advisable to plot quantities with significantly different error magnitudes on the same graph. In Fig. 1-3, the errors for ΔE_t and $\Delta\rho$ are lost among other quantities. While this representation can show which quantities have more dominant relative errors, it might make interpreting the correlations a bit difficult. More comprehensible figures would be more valuable.

AR: *We thank the reviewer for this valuable comment. The primary purpose of Fig. 1-3 was to show the reader the general trends in all errors and hybrid functionals. Due to the large number of XC hybrids, another way of presenting these data is very troublesome. The correlation plots (Fig. 4-6 and Fig. S4-S7) are supposed to show the general trends attributed to the groups of functionals, whereas Tables 1 and 2 focus on the worst and best XC hybrid functionals. We agree, however, that the ΔE_t and $\Delta\rho$ errors are much smaller and more complex to interpret in this scale. Thus, to make the presentation of energy and density errors more understandable, we present errors on total energy and density on an additional separate plot (Bottom panel of Fig. 3).*

RC: 2. It is commendable that the authors separately examined the correctness of the XC potential in the core and valence regions. It would be important to mention for which properties the accurate description of these regions is crucial (e.g., atomization energies) and for which properties it is less critical (e.g., long-range interactions).

AR: *We appreciate this comment. This kind of analysis is indeed very useful since many XC hybrids (especially global hybrids) have wrong asymptotic behavior of XC potential; hence, it is not simple to judge the overall quality of potential with one error indicator. As is commonly known, the quality of XC potential in asymptotic regions directly transfers to the quality of first ionization potential and excited state energies. This is visible, for example, in the cases of long-range corrected hybrids. On the other hand, this region is energetically evanescent, which is irrelevant for most ground-state properties. In turn, the quality of XC potential in the core region, which is measured by $\Delta\rho^{ref}v_{xc}$ indicator, is related to the quality of electron density that was investigated in our group (see J. Chem. Phys. 135, 114111 (2011), https://doi.org/10.1063/1.3636114, and Molecular Physics, 112(5–6), 700–710, https://doi.org/10.1080/00268976.2013.854424). However, the direct correlation between v_{xc} and ground state properties has never been investigated, and currently, it is beyond the scope of the current investigation. We note that energetics is mainly governed by the quality of XC functional, which is dominated by functional-driven error, as shown in Fig. S4. Thus, to investigate*

the correlation between the quality of v_{xc} in the core region with the ground state properties one would require the evaluation of these quantities on the set of well define ab inito WFT densities as used e.g. in 10.1063/1.3636114.

We have added a sentence on page 6:

"We recall that the asymptotic behavior of XC potential has a significant impact on the quality of first ionization potential as well as excitation energies[19,42,43]."

RC: 3. If possible, the authors could compare the performance of the best functionals with the results obtained for the GMTKN55 benchmark set if those are available. It would be interesting to see how the results for various physical and chemical properties correlate with the results discussed here (see the previous question).

AR: *We thank the reviewer for this comment. We recall that the main aim of the current work was to present a new methodology to evaluate the quality of DFT functionals (hybrid in our case) based on very fundamental quantities, i.e., XC potentials, self-consistent electron densities, first ionization potentials (IP), and total energies and presenting some interesting correlations between them. We think that the inclusion in the discussion of the energetically based GMTKN55 benchmark will change or even shift the scope of the paper, which currently emphasizes the crucial role of the aforementioned fundamental quantities. Moreover, we would like to underlay that to reasonably discuss how the results for various physical and chemical properties correlate with those discussed here; one would need to compute all main quantities for 2462 single-point calculations available in the GMTKN55 benchmark. This would be incredibly challenging for WY inverse methods where the proper choice of basis set is crucial for obtaining reliable* v_{xc} *potentials. From our point of view, this kind of analysis would be very valuable. However, it would require much more time to make some relevant conclusions. Nonetheless, we think the suggestion pointed out here and in p. 2 would be an excellent topic for a separate study, which we will definitely pursue.*

RC: 4. I am interested in the authors' opinion on the following sentence: "To summarise our main findings in one sentence, we can state that we presented clear evidence that the vast majority of non-empirical DHDFs do not live up to the initial hopes that some in the developer community may have had [doi: 10.1039/c8cp03852j]." The authors' opinion in the cited manuscript is that only the final numerical results matter. It might be worthwhile to discuss this thought in detail in the present manuscript, considering whether the authors agree or disagree with this opinion.

AR: *Thank you for this "provocative" comment. From our point of view, this question is the old "standard" ongoing problem between theory, empiricism, and pragmatism in science, and it is essential right now in the application era. It happens many times that semi-empirical methods work better (in general picture) than theoretically justified methods. This also occurs in the wave function theory, e.g., CCSD(T) vs. CCSD[T]. In the case mentioned in the comment DH DFAs, this shows that non-empirical DH still needs more understanding and additional work, e.g., the construction of better, more accurate semi-local DFAs used in the construction of DH DFAs. As to the question regarding "the authors" opinion about the cited manuscript that only the final numerical results matter." Here we have an unambiguous opinion - there must always be "something" behind the numbers - we have to understand methods and be able to answer the question "why they work?". Theory gives us tools to understand their construction. We think that computational methods must be predictable, and the users must understand what is hidden in the "black box" tools. This is also one of the reasons we included exchange-correlation potentials in our evaluation, which can help overcome current DFA problems and bring new ways for new functional development.*

RC: 5. The authors examined the obtained results depending on the grid. It might be interesting to see whether using different quadratures, such as Mura–Knowles, Gauss–Chebyshev, Treutler–Ahlrichs, and Euler–Maclaurin, affects the results. I guess that it does not influence the results too much, but I would be interested to know if the authors have any experience with this.

AR: *Yes, we checked a few different quadratures implemented in the PySCF package at the initial project stage, i.e., Treutler, Delley, and Mura-Knowles. This is presented in Fig. 1 in the reply for the* He_2 *molecule with FCI density. In the case of* $\Delta\rho$ *and* $\Delta\rho^{ref}v_{xc}$ *errors, there is no difference in the behavior of errors. For* Δv_{xc}*, in turn, we see only small quantitative changes in error values, which do not affect our findings and conclusions (the qualitative picture remains the same); we decided to use the default quadrature in the PySCF program, i.e., the Treutler one. This behavior should not be surprising since most quadratures are optimized for general numerical evaluation of basis functions, electron densities,* v_{xc} *matrix elements, and not* v_{xc} *potential on the*

grid. The radial mesh can probe the asymptotic region differently, leading to the differences in Δv_{xc} error. Nonetheless, the qualitative picture of all indicators remains very similar despite the quadrature used.

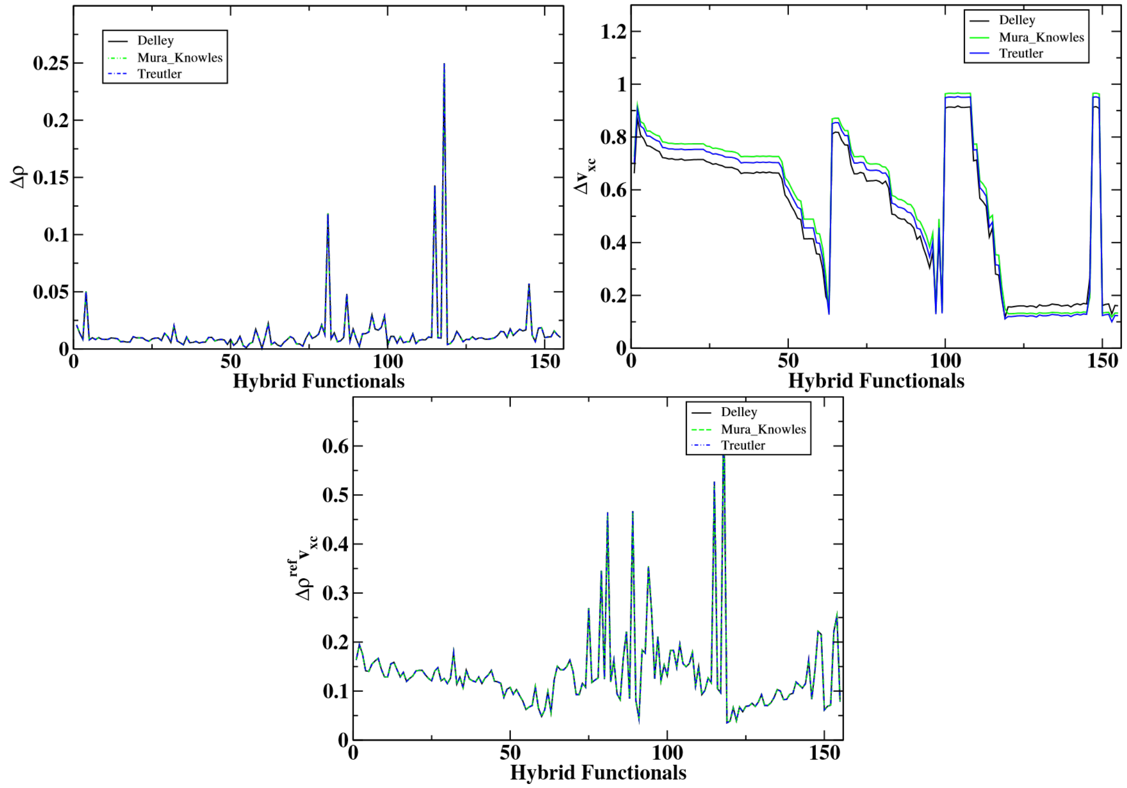

Figure 1: Impact of quadrature choice on the quality of the error indicators for the He_2 molecule. Errors are calculated for the FCI reference quantities.

RC: 6. The procedure can be easily generalized for double hybrid functionals as well if I understand correctly. Due to local MP2 implementations, the use of these functionals may become increasingly dominant in the community. Do the authors plan to investigate these functionals as well

AR: *Indeed, the same procedure can be applied to investigate the double hybrid (DH) family of functionals and any other KS DFA. However, one must remember that at the v_{xc} level, the DH DFA is reduced to a hybrid (the second-order contribution is disregarded in the v_{xc} potential). Thus, a full investigation of potential would require employing the optimized effective potential method at second order. We plan to evaluate the double hybrid functionals in a slightly different context.*

Reviewer #2

RC: 1 - molecules of interest in this work are very small and thus without chemical significance in comparison to the ones belonging for instance to the GMTKN55 dataset.

AR: *We agree that the GMTKN55 dataset contains a much broader spectrum of cases important for chemistry. However, inclusion in our benchmark sets of small molecules has a few reasons. The first one is very practical: the inverse methods we use (or any other available) for calculating XC potentials from the density can not be applied to large systems with such accuracy because the inverse procedures are numerically very challenging. Here, we are also limited to systems where very accurate FCI or CCSD(T) densities can be computed. We recall that similar sizes of systems have also been considered in other studies regarding*

fundamental aspects of DFT (see, e.g., Science355,49-52(2017).DOI:10.1126/science.aah5975 or JCTC 2017 13 (10), 4753-4764 DOI: 10.1021/acs.jctc.7b00550). The second reason is more fundamental - evaluation and analysis of the KS
DFT functionals for small systems is crucial for the correctness of the construction of new XC approximation. If there is a problem for small systems, it cannot be ignored only because we do not see it for a larger one. And finally, the benchmark contains important molecules, e.g., astrochemistry, ultracold chemistry.

RC: 2 - Figures 2 and 3 are hard to analyze for specialists. We can imagine the difficulty for non specialists. A graph function of the fraction exact-like exchange would be much more explicit, as well as focusing on the performance of commonly used hybrids in literature.

AR: *We thank the reviewer for this comment. To facilitate the reader's interpretation of these graphs, we have provided clear guidelines for the reader to interpret those figures at the beginning of the "Computational Methods" section. As pointed out by the reviewer, the fraction of exact-like exchange was utilized as one of the indicators to arrange the XC hybrid's performance.*

To clarify this fact, we have added the sentence on page 4.

"The same ordering of DFAs is utilized in Fig1 and Fig2 "

RC: 3 - it is not clear to me if the error measurements are computed via reference densities or self-consistent densities. Usually, errors are derived from self-consistent computations. What is here the incidence?

AR: *We thank the reviewer for this comment. We agree that this was not clearly stated in the manuscript. All quantities, except v_{xc}, have been obtained from self-consisted GKS hybrid calculations. The v_{xc} for a given hybrid DFA, in turn, was obtained via the Wu-Yang inverse procedure starting from the self-consisted GKS density matrix. We have made a few amendments in the text to clarify this.*

RC: 4 - it is a pity that the authors did not compare their approach with existing benchmarks focusing on energy and density errors.

AR: *We thank you for this comment. However, we think that using different existing sets to benchmark the energy and density errors separately for all functionals could lead to biased conclusions. We underlay that, e.g., in the case of the GMTKN55 benchmark set, the investigation would require computing all main quantities for 2462 single-point calculations available in the GMTKN55 benchmark for 155 hybrid DFAs. This would be very challenging for the reasons mentioned in p. 1.*

RC: 5 - Typos: p.5 Hohengerg-Kohn

AR: *We have corrected the typo in the revised manuscript.*
